# The phosphatase PRL-3 affects intestinal homeostasis by altering the crypt cell composition

**DOI:** 10.1007/s00109-021-02097-9

**Published:** 2021-06-15

**Authors:** Teresa Rubio, Judith Weyershaeuser, Marta G. Montero, Andreas Hoffmann, Pablo Lujan, Martin Jechlinger, Rocio Sotillo, Maja Köhn

**Affiliations:** 1 European Molecular Biology Laboratory, Genome Biology Unit, Heidelberg, Germany; 2 Present Address: Institut d’Investigació Biomèdica de Bellvitge (IDIBELL), Hospital Duran i reynals, L’Hospitalet de Llobregat, Spain; 3 Signalling Research Centers BIOSS and CIBSS, Albert-Ludwigs-Universität Freiburg, Freiburg, Germany; 4 Faculty of Biology, Albert-Ludwigs-Universität Freiburg, Freiburg, Germany; 5 European Molecular Biology Laboratory Rome, Adriano Buzzati-Traverso Campus, Via Ramarini 32, Monterotondo, Italy; 6 European Molecular Biology Laboratory, Cell Biology and Biophysics Unit, Heidelberg, Germany; 7 Present Address: ICFO-Institut de Ciencies Fotoniques, The Barcelona Institute of Science and Technology, Castelldefels, Spain; 8 Present Address: MOLIT Institut GmbH, Heilbronn, Germany; 9 Division of Molecular Thoracic Oncology, Deutsches Krebsforschungszentrum (DKFZ), Heidelberg, Germany

**Keywords:** PRL-3, PTP4A3, Lgr5, Intestinal stem cells, Paneth cells, Organoids, Stem cell homeostasis, Phosphatase, Cancer

## Abstract

Expression of the phosphatase of regenerating liver-3 (PRL-3) is known to promote tumor growth in gastrointestinal adenocarcinomas, and the incidence of tumor formation upon inflammatory events correlates with PRL-3 levels in mouse models. These carcinomas and their onset are associated with the impairment of intestinal cell homeostasis, which is regulated by a balanced number of Paneth cells and Lgr5 expressing intestinal stem cells (Lgr5+ ISCs). Nevertheless, the consequences of PRL-3 overexpression on cellular homeostasis and ISC fitness in vivo are unexplored. Here, we employ a doxycycline-inducible PRL-3 mouse strain to show that aberrant PRL-3 expression within a non-cancerous background leads to the death of Lgr5+ ISCs and to Paneth cell expansion. A higher dose of PRL-3, resulting from homozygous expression, led to mice dying early. A primary 3D intestinal culture model obtained from these mice confirmed the loss of Lgr5+ ISCs upon PRL-3 expression. The impaired intestinal organoid formation was rescued by a PRL inhibitor, providing a functional link to the observed phenotypes. These results demonstrate that elevated PRL-3 phosphatase activity in healthy intestinal epithelium impairs intestinal cell homeostasis, which correlates this cellular mechanism of tumor onset with PRL-3-mediated higher susceptibility to tumor formation upon inflammatory or mutational events.

**Key messages**

• Transgenic mice homozygous for PRL-3 overexpression die early.

• PRL-3 heterozygous mice display disrupted intestinal self-renewal capacity.

• PRL-3 overexpression alone does not induce tumorigenesis in the mouse intestine.

• PRL-3 activity leads to the death of Lgr5+ ISCs and Paneth cell expansion.

• Impairment of cell homeostasis correlates PRL-3 action with tumor onset mechanisms.

**Supplementary Information:**

The online version contains supplementary material available at 10.1007/s00109-021-02097-9.

## Introduction

PRL-3 (PTP4A3) belongs to the subfamily of the PRL proteins comprising three members (PRL-1, PRL-2, and PRL-3). While PRL-1 and PRL-2 are expressed in healthy human adult tissue, PRL-3 is barely found, but it is highly expressed in primary tumors of different origin and metastases, correlating with poor outcome [1–3]. PRL-3 activity was shown to promote tumor growth and metastasis formation in orthotopic mouse tumor models [4, 5] and also cell migration, invasion, and epithelial architecture disruption [1, 3, 6–10]. A *PTP4A3* knockout mouse model was shown to be grossly normal, supporting non-essential roles for PRL-3 in healthy tissue [11]. These mice had a 50% reduced incidence of colon cancer formation when triggered by inflammation through the chemotoxins azoxymethane (AOM) and dextran sodium sulfate (DSS), reflecting a potential role for PRL-3 in cancer initiation [11]. In line with this study, a gain-of-function PRL-3 transgenic (TG) mouse model showed higher incidence of colon malignancy upon DSS treatment [12]. Nevertheless, within the 8 weeks of PRL-3 overexpression in this mouse model without DSS treatment, no cancer development was observed [12]. Whether there are cellular consequences of PRL-3 expression in vivo that could potentially prime the intestinal tissue for cancer initiation, as suggested by the results from the DSS-induced inflammation, and whether the expression of PRL-3 alone over a longer period of time leads to cancer development have not yet been addressed. Thus, it is critical to further evaluate the consequence of PRL-3 expression in a gain-of-function mouse model system.

Colorectal cancer (CRC) has been associated with the malignant transformation of intestinal stem cells (ISCs) and ISC homeostasis failure [13]. The proliferation of ISCs is controlled through Wnt signaling [14–18]. The receptor of R-spondin Lgr5 is an agonist of Wnt signaling [19, 20] and a crypt SC marker [21]. Wnt ligands are produced in the crypts by the epithelial Paneth cells [19], which also require a strong Wnt signal for their differentiation [14, 22], and the mesenchyme, which also produces R-spondin [23]. In the adult intestinal epithelium, ISCs are also important for intestinal cell homoeostasis and for regeneration after a lesion [24, 25]. In order to accomplish these functions, they replace each other in a stochastic fashion in order to keep a fixed number of ISCs in the crypt. As a consequence of this model, ISCs are not long-lived SCs, and, moreover, the progeny of a single SC is able to displace all other SCs from the niche [26, 27]. It was hypothesized that cells that have acquired a mutation would be likely replaced by their neighboring wt ISCs to avoid the fixation of the mutation. Accordingly, in a cancer background, the reduction of Lgr5+ ISCs per crypt facilitated the rapid fixation of Apc tumor suppressor-deficient ISCs in the crypts resulting in an accelerated tumorigenesis [28]. Dysfunctional or reduced crypt homeostasis has been accompanied by a decrease in ISC numbers and Paneth cell expansion. This phenomenon has been associated with inflammatory bowel disease, with the administration of the methotrexate rheumatoid arthritis treatment to rats, after intestinal resection, and with the response to mucosal damage as well as to doxorubicin [14, 29–34]. Considering that (i) inflammatory events affect crypt cell homeostasis, that (ii) reduced renewal capacity of the intestine contributes to cancer formation, and that (iii) PRL-3 expression correlates with tumor formation upon inflammatory events in the intestine, we aim here to answer whether PRL-3 expression has an influence on crypt homeostasis. To this end, we generate a doxycycline (dox)-inducible PRL-3 overexpressing mouse line to investigate the consequences of PRL-3 overexpression on cellular homeostasis and ISC fitness in vivo and to answer whether PRL-3 overexpression by itself causes tumor formation in this system over a longer period of time*.*

## Results

### Homozygous PRL-3 transgenic overexpressing mice have a short lifespan

To assess the physiological consequences of PRL-3 overexpression in vivo, we generated C57BL/6J PRL-3 TG dox-inducible mice. Unlike the previously reported model [12], we used a *hemagglutinin* (*HA*) tagged human *Ptp4a3* cDNA and introduced it in the collagen type 1 (colA1) locus avoiding random transgenesis [35]. In this system, the embryonic stem (ES) cells used have been previously engineered to express a tetracycline-inducible rtTA transactivator driven by the endogenous Rosa 26 promoter (R26-rtTA) to achieve ubiquitous expression (Fig s1A). Western blot analysis of different ES cell clones confirmed the expression of HA-PRL-3 upon dox addition (Fig s1B and Supp. Material 1). Two HA-PRL-3 independent transgenic lines were obtained and characterized—FA1 and FB1 (“Methods” section).

**Fig. 1:**
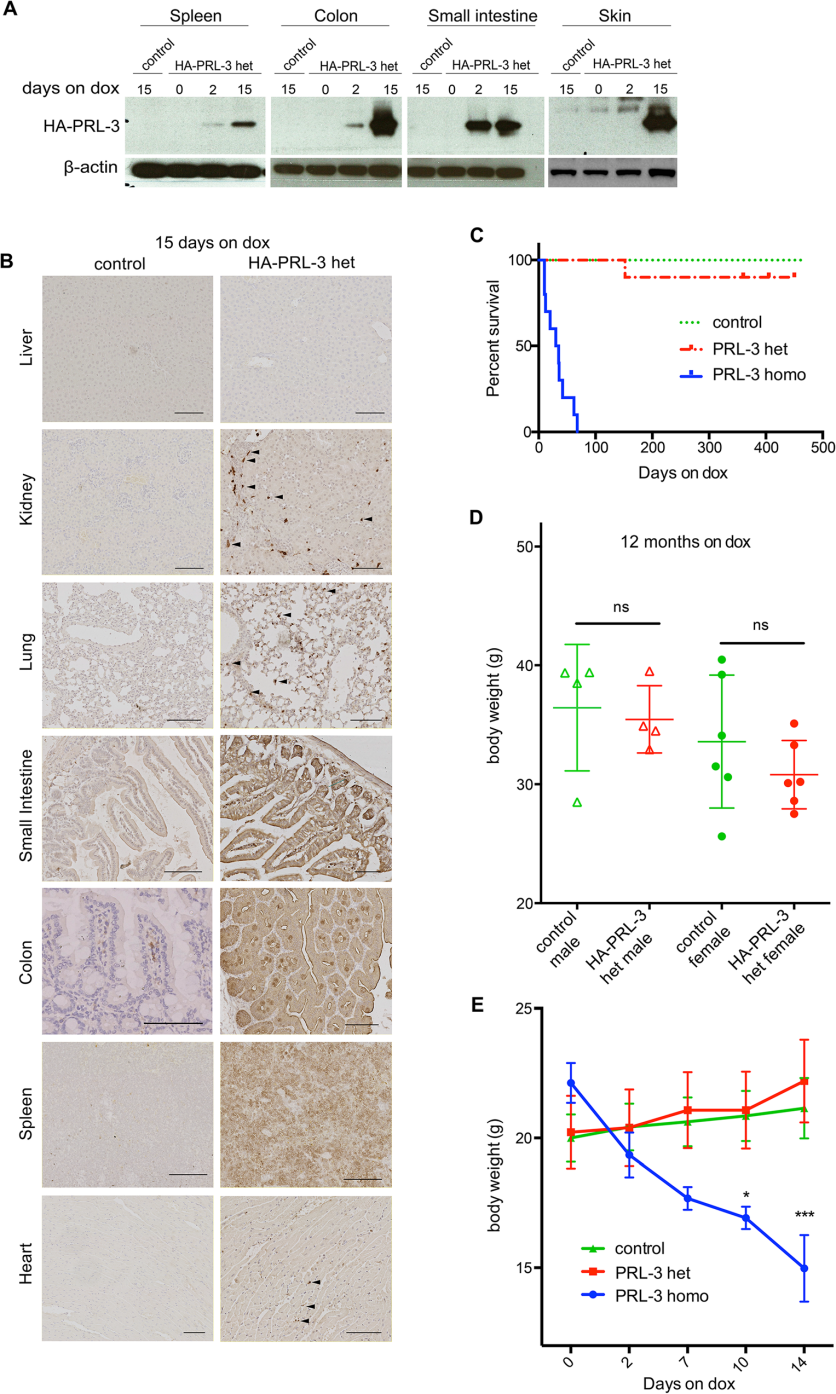
Characterization of PRL-3 TG mice (FA1 colony). **a** Western blot analysis of tissue-derived samples from HA-PRL-3 wt/R26-rtTA het (control) mice treated 15 days with dox and HA-PRL-3 het/R26-rtTA het (HA-PRL-3 het) mice on dox for 0 (not treated), 2, or 15 days. HA signal was detected using specific antibodies. Actin was used as a loading control. HA expression was found in the spleen, skin, colon, and small intestine. **b** Anti HA immunohistochemical staining of different tissue sections derived from control or HA-PRL-3 het mice 15 days on dox. Scale bar = 100 μm. **c** Kaplan-Meier survival curve on doxycycline food for HA-PRL-3 het/R26-rtTA het (PRL-3 het), HA-PRL-3 homo/R26-rtTA het (PRL-3 homo), and HA-PRL-3 wt/R26-rtTA het (control) mice. N=10 mice for each genotype. HA-PRL-3 homo mice show a shorter lifespan compared with control and HA-PRL-3 het mice. **d** Body weight split by gender N=4 for male and N=6 for females for each genotype. HA-PRL-3 wt/R26-rtTA het (control) and HA-PRL-3 het/R26-rtTA het (HA-PRL-3 het) mice, fed with dox for 12 months, show no differences in body weight (g=grams). Data represents mean ± s.d., differences non-significant (ns) with one-way ANOVA with Tukey’s multiple comparison test. **e** Weight measurements (in grams) of PRL-3 homo, PRL-3 het, and control mice 2 weeks on dox food showed a decrease in the weight of HA-PRL-3 homo compared to control and HA-PRL-3 het mice. Data represent mean ± s.d. N=5. *P≤0.1, ***P≤0.001 ordinary two-way ANOVA with Tukey’s multiple comparison test

Upon 15 days of dox treatment, PRL-3 mRNA was detected in all the tested organs derived from bi-transgenic R26-rtTA and HA-PRL-3 heterozygous (het) mice (Fig s1C). Moreover, PRL-3 overexpression was strongly detected in colon, small intestine (SI), spleen, and skin by western blotting in a time-dependent manner, but not in all other organs tested (Fig. 1a, Fig s1D and Supp. Material 1). Mice containing only one transgene, either HA-PRL-3 or R26-rtTA, were used as controls. Immunohistochemical staining validated PRL-3 expression in those organs and also found small populations of cells expressing PRL-3 in the kidney, lung, and heart (Fig. 1b).

Heterozygous bi-transgenic HA-PRL-3 het/R26-rtTA het mice did not exhibit evident developmental defects, structural abnormalities, lifespan variation, differences in body as well as organ weight, or tumor development after 12 months of dox treatment neither in FA1 (Fig. 1c, d and Fig s1E, F) nor in FB1 (Fig s1G, H, I) compared to the HA-PRL-3 wt/R26-rtTA het control mice. Given the identical results for both transgenic lines, the following studies were carried forward using the FA1 transgenic line. Interestingly, the HA-PRL-3 insertion in homozygosis (HA-PRL-3 homo) led to a shorter lifespan (15–30 days) compared to HA-PRL-3 het and control mice upon dox treatment (Fig. 1c). Nevertheless, the lifespan was the same for all three genotypes (PRL-3 wt, het, and homo) in the absence of dox (Fig s1K) confirming that the observed phenotype is due to PRL-3 expression and not due to any alteration in the ColA1 expression. In addition, this short lifespan observed in HA-PRL-3 homo mice correlated with a striking decrease in their body weight (Fig. 1e).

### HA-PRL-3 aberrant expression leads to a decrease of Lgr5+ ISCs and an increase of Paneth cell counts in the intestinal crypts

**Fig. 2:**
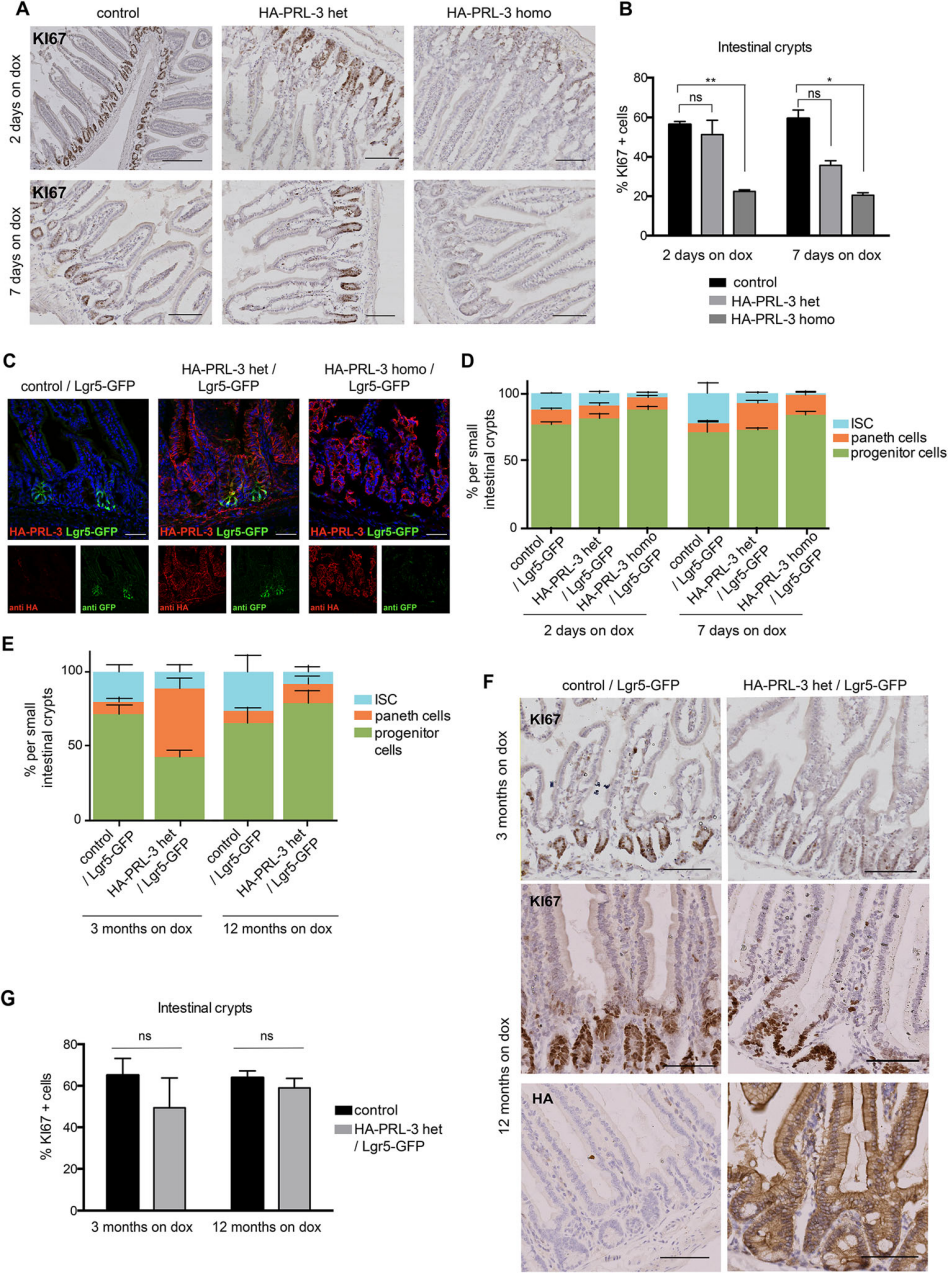
Characterization of the intestinal crypts of PRL-3 TG mice. **a** Representative images of KI67 immunohistochemistry of small intestine biopsies derived from HA-PRL-3 wt/R26-rtTA het (control), HA-PRL-3 het/R26-rtTA het (HA-PRL-3 het), and HA-PRL-3 homo/R26-rtTA het (HA-PRL-3 homo) mice fed with dox food for 2 days or 7 days. Scale bar: 100 μm. **b** Percentage of KI67-positive cells (relative to total cells counted) in the intestinal crypts from the different genotypes represented in (**a**). 1000 cells were analyzed per tissue sample. Values represent mean ± s.d. of three biological replicates. Statistics: *P≤0.1, **P≤0.01 one-way ANOVA test with Dunnett’s multiple comparisons, compared to control mice. **c** Overexpression of PRL-3 (7 days on dox) leads to a decrease of Lgr5-GFP-positive cells. Representative IF images of small intestine tissue samples from control/Lgr5-GFP, HA-PRL-3 het/Lgr5-GFP, and HA-PRL-3 homo/Lgr5-GFP using anti-HA antibody (red) for HA-PRL-3 detection and anti-GFP antibody (green) for Lgr5-GFP detection. Bottom: individual channels. Scale bar 50 μm. N= 3 for each genotype. **d**, **e** Frequencies of sorted ISC Lgr5-GFP high, progenitors Lgr5-GFP low, and Paneth cells Lgr5-GFP –/CD24 high/Sidescatter high from the entire small intestine of control/Lgr5-GFP, HA-PRL-3 het/Lgr5-GFP and HA-PRL-3 homo/Lgr5-GFP mice, treated with dox 0, 2, or 7 days (**d**) and 3 or 12 months (**e**). The statistics are as follows: **d** ISCs 2 days on dox: * PRL-3 het vs PRL-3 homo, PRL-3 het vs control ns and ** PRL-3 homo vs control. Progenitors and Paneth cells 2 days on dox: ns. ISCs 7 days on dox: PRL-3 het vs PRL-3 homo: ns, * PRL-3 het vs control, ** PRL-3 homo vs control. Progenitors 7 days on dox: ns between PRL-3 homo, het and control. Paneth cells 7 days on dox: *** PRL-3 het vs control, **PRL-3 homo vs control, PRL-3 het vs PRL-3 homo ns. **e** 3 months on dox PRL-3 het vs control: ISCs ns, progenitors * and Paneth cells **. 12 months on dox PRL-3 het vs control: ISCs, progenitors and Paneth cells ns. Bars represent mean ± s.d. of four biological replicates. ns, not significant; *P≤0.1,**P≤0.01,***P≤0.001; ordinary two-tailed one-way ANOVA with Tukey’s multiple comparison test. **f** Representative images of KI67 staining of small intestine biopsies from the mice used in (**e**), upper panel 3 months dox treated mice, middle panel samples from 12 months dox treated mice and lower panel HA staining for PRL-3 detection in mice after 12 months on dox food. **g** Percentage of KI67 positive cells (relative to total cells counted) in the intestinal crypts in the different conditions represented in (**f**). 1000 cells were analyzed per tissue sample. Values represent mean ± s.d. of three biological replicates. Statistics: *P*≤0.1 *t*-test, compared to control mice

The epithelium of the SI is a highly proliferative tissue maintained by Paneth cells and Lgr5+ ISCs located in the crypts. A defect of Paneth and ISC homeostasis has been associated with more sensitiveness to tumor development through easier fixation of sporadic cancer ISC transformations [28]. Because the SI mimics the physiology of the colon and it is easier to handle with respect to ex vivo 3D cell culture systems, for a better comparison of the in vivo and following in vitro experiments, we focused our attention here on the SI tissue. Thus, we tested whether PRL-3 overexpression interferes in the self-renewal ability of intestinal tissue by analyzing cell proliferation. Intestinal tissue samples derived from control HA-PRL-3 het or HA-PRL-3 homo mice treated up to 7 days with dox (due to the short lifespan of HA-PRL-3 homo mice) were stained with the KI67 proliferation marker. As expected, no proliferation was observed in the villi of any of the mice (Fig. 2a). However, a dramatic decrease in the number of KI67+ cells was observed in the intestinal crypts derived from HA-PRL-3 homo mice compared to controls, while no significant differences were seen between HA-PRL-3 het and controls (Fig.2a, b). In light of this result, we hypothesized that HA-PRL-3 overexpression could affect specifically the ISCs, which sustain intestinal cell proliferation and tissue self-renewal, in a dose-dependent manner.

To gain further insights, HA-PRL-3 TG mice were crossed with Lgr5-EGFP-IRES-CreERT2 knock-in mice [21] to obtain HA-PRL-3/R26-rtTA/Lgr5-EGFP-IRES-CreERT2 (named HA-PRL-3/Lgr5-GFP) mice in order to tag the Lgr5+ ISCs with GFP. After dox treatment, intestinal biopsies derived from this new mouse strain were stained for immunofluorescence against HA (for HA-PRL-3 detection) and GFP (for Lgr5-GFP detection), and the nuclei were stained with Hoechst. HA-PRL-3 homo/Lgr5-GFP showed less Lgr5-GFP signal compared to intestinal tissue derived from HA-PRL-3 wt and het mice (Fig 2c), suggesting that PRL-3 overexpression leads into the reduction of ISC numbers. This observation was quantified by flow cytometry (FC) where the amount of Lgr5+ ISCs derived from the HA-PRL-3 het/Lgr5-GFP was already significantly decreased upon dox treatment compared to those derived from HA-PRL-3 wt/Lgr5-GFP control mice (Fig. 2d). Interestingly, when HA-PRL-3 was present in homozygosis, the Lgr5+ ISCs were almost all depleted, suggesting that PRL-3 impairs ISC numbers in a dose-dependent manner and, consequently, explaining the reduced lifespan of the homozygous mice. To this end, we confirmed the higher expression of HA-PRL-3 in homozygous compared to heterozygous mice in SI tissue by western blot (Fig S2A, B and Supp. Material 1). Previously, van Es et al. showed that the decrease of Lgr5+ ISCs in intestinal crypts was linked to an increase of Paneth cells [22]. In line with this, we also observed that the decrease in Lgr5+ ISC cells upon PRL-3 overexpression correlated with increased number of Paneth cells after 7 days of dox treatment (Fig. 2d).

Next, we investigated the long-term consequences of PRL-3 overexpression in the intestinal epithelium. We measured the different crypt cell populations by FC in intestine samples from mice treated for 3 or 12 months with dox. HA-PRL-3 homo mice were not possible to evaluate under these conditions due to their short lifespan. Keeping the tendency observed at 7 days on dox, the number of Paneth cells was increased in HA-PRL-3 het/Lgr5-GFP mice after 3 months, while ISCs kept the same proportion as after 7 days on dox (Fig. 2e). We hypothesized that the Paneth cell numbers were increased in order to compensate for the mild decrease in ISC numbers observed in the HA-PRL-3 het mice, taking over their role as proliferative cells early on. After 12 months on dox, the number of ISCs in HA-PRL-3 het/Lgr5-GFP mice was constant compared to the number after 3 months and again lower than for the control mice. The Paneth cell number was reduced in HA-PRL-3 het compared to earlier time points but still slightly higher than in the control population. At this time point, the organism might have adapted to the lower ISC numbers, reducing the number of Paneth cells compared to earlier time points. This could explain why the heterozygous mice survived longer than the homozygous, where the ISCs were almost depleted already at 7 days on dox, not giving the Paneth cells enough time for the compensation. In line with the Paneth cells early taking over the proliferative role and the later organismal adaptation, no differences were found in the proportion of proliferative cells (KI67+) in intestinal biopsies derived from the mice used in Fig. 2e by KI67 immunohistochemical staining (Fig. 2f, g; see Fig. S2C, D for colon tissue confirming a similar phenotype), even though HA-PRL-3 expression was confirmed after 12 months of dox treatment (Fig. 2f). Altogether, this shows that HA-PRL-3 overexpression in heterozygosis in vivo led to a decrease of Lgr5+ ISCs, which was likely compensated by increasing the Paneth cell numbers to maintain intestinal cell homeostasis and stemness. The phenotype observed in the intestinal epithelium is PRL-3 dose-dependent since homozygous HA-PRL-3 mice have completely lost all the Lgr5+ ISCs, likely leading to the early death of these mice.

### PRL-3 overexpression leads to aberrant intestinal organoid formation

**Fig. 3:**
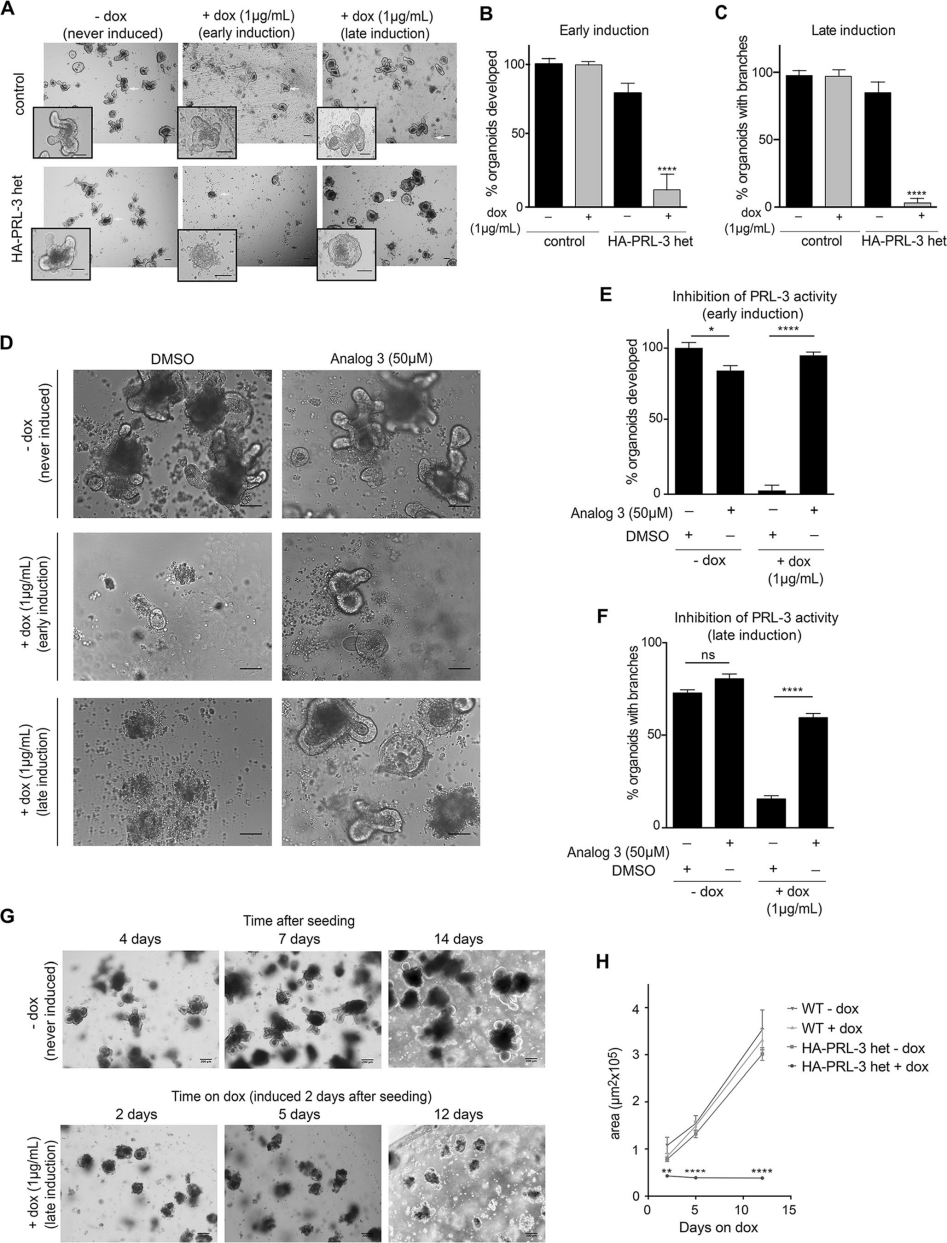
PRL-3 overexpression leads to aberrant intestinal organoid formation. **a** Representative bright field inverted microscopy images of small intestinal 3D cell cultures derived from HA-PRL-3 wt/R26-rtTA het control mice and HA-PRL-3 het/R26-rtTA het mice. HA-PRL-3 expression was induced in vitro with 1μg/mL of dox. First column: no dox treatment (never induced); second column: dox was added in the media at the same time of the cell seeding (early induction); and third column: dox was added 2 days after cell seeding when the organoids were already formed (late induction). Pictures were taken after 4 days in culture for all conditions. Scale bar: 100 μm. **b** Percentage of organoids developed in each condition at early induction of PRL-3 overexpression relative to the never induced control. **c** Percentage of normal organoids with branches, developed after late induction of PRL-3 overexpression calculated relative to the total number of organoids developed in each condition. **d** Selected bright field inverted microscopy images of the small intestinal organoids derived from HA-PRL-3 het mice never induced or late induced with dox, after 4 days in culture, treated with DMSO or with 50 μM of *analog 3*, a PRL phosphatase inhibitor, which was added to the culture media at the same time as dox. **e** Percentage of organoids developed for each condition in (**d**) at early HA-PRL-3 induction, calculated relative to the number of organoids developed in the control never induced with DMSO treatment. **f** percentage of organoids with branches for each condition in (**d**) at late induction calculated relative to the total organoids. **b**, **c**, **e**, **f** Data are mean ± s.d. N=3. ns not significant, *P≤0. 1, ****P≤0.0001, ordinary two-tailed one-way ANOVA with Tukey's multiple comparison test. **G** Representative bright field inverted microscopy images of small intestine organoids from HA-PRL-3 het mice, never induced as control or late induced (after 2 days in culture). Pictures were taken after 2, 5 or 12 days of dox treatment. Scale bar: 200 μm. **h** Representation of the average area of the HA-PRL-3 het organoids +/- dox shown in (**g**), as well as of wt organoids +/- dox as control (images not shown). Values represent the mean ± s.d. N=75*.* **P≤0.01, ****P≤0.0001 ordinary two-tailed two-way ANOVA with Tukey's multiple comparison test

To better understand the consequences of high PRL-3 levels in vivo, we used the capacity of ISC-containing epithelial crypts to form clonogenic organoids [36, 37] as an in vitro assay of intestinal self-renewal potential. To this end, isolated crypts from untreated HA-PRL-3 het/R26-rtTA mice were cultured embedded in extracellular matrix (see “Methods”), and PRL-3 expression was then induced in vitro at two different time points: at the moment of the cell seeding (early induction) in order to analyze the effect of HA-PRL-3 overexpression in tissue development and 2 days after seeding (late induction) when the organoids are already formed, to mimic the overexpression of PRL-3 in an adult intestine. Interestingly, organoids failed to form when PRL-3 overexpression was early induced (Fig. 3a, b), implicating that PRL-3 expression is toxic for Lgr5+ SCs, which are the basis for the development of intestinal organoids [37]. Furthermore, when PRL-3 expression was induced 2 days after seeding of the organoids (late induction), they were transformed into aberrant intestinal organoids lacking the crypt-like domain (branches) where ISCs reside (Fig. 3a right panel and Fig. 3c). This phenotype was the same in organoids derived from the SI of homo HA-PRL-3 expressing mice and was also identical in organoids derived from colon of HA-PRL homo and het mice (Fig S3A, B). Furthermore, we confirmed by western blot that under these conditions, HA-PRL-3 was expressed in organoids derived from SI and colon, with higher expression in HA-PRL-3 homo organoids (Fig S3C-F and Supp. Material 1). These results reflect the strong phenotype of HA-PRL-3 homozygous expression in vivo.

Subsequently, we investigated whether the observed phenotype in the intestinal organoids upon PRL-3 overexpression was due to its phosphatase activity. With this aim, PRL-3 overexpressing organoids were cultured in presence of *analog 3 (A3)*, a PRL inhibitor that was shown to be active and non-toxic in 3D cell cultures of laboratory cell lines [9, 38]. The inhibitor was added at the same time as dox to inhibit PRL activity in parallel to PRL-3 overexpression in order to avoid residual phosphatase activity. As expected from the results in Fig. 3a–c, DMSO-treated organoids failed to form when HA-PRL-3 expression was early induced with dox and showed a round shape when HA-PRL-3 expression was induced after 2 days of seeding (late induction) (Fig. 3d–f). However, when these organoids were treated together with *A3*, the percentage of formed organoids at early HA-PRL-3 induction increased (Fig. 3d, e), as did the percentage of organoids with branches for the late induction (Fig. 3d, f). *A3* treatment alone in uninduced organoids appeared to be mildly cytotoxic in early induced (Fig. 3e) but not in late induced (Fig. 3f) organoids. Together, these results confirmed that the observed phenotype depended on PRL-3 phosphatase activity.

This characteristic phenotype can be due to a morphological transformation of the epithelial structure or due to the death of the Lgr5+ ISCs (located in the branches). In order to discern between these two possibilities, the area of these cultures after late induction was monitored over 12 days. As shown in Fig. 3g, h, PRL-3 overexpressing organoids (HA-PRL-3 het/R26-rtTA het) were not able to grow further upon dox treatment compared to the never induced or the HA-PRL-3 wt/R26-rtTA het control organoids treated or not with dox, suggesting that PRL-3 overexpression provoked organoid death. This result was validated by a cell viability propidium iodide (PI) assay where HA-PRL-3 overexpressing intestinal organoids showed less live cells compared to controls where PRL-3 was not expressed (Fig S4A). Taken together, these results correlate with the failure in organoid formation when HA-PRL-3 overexpression was induced at an earlier stage.

### PRL-3 overexpression leads to the death of ISCs

**Fig. 4:**
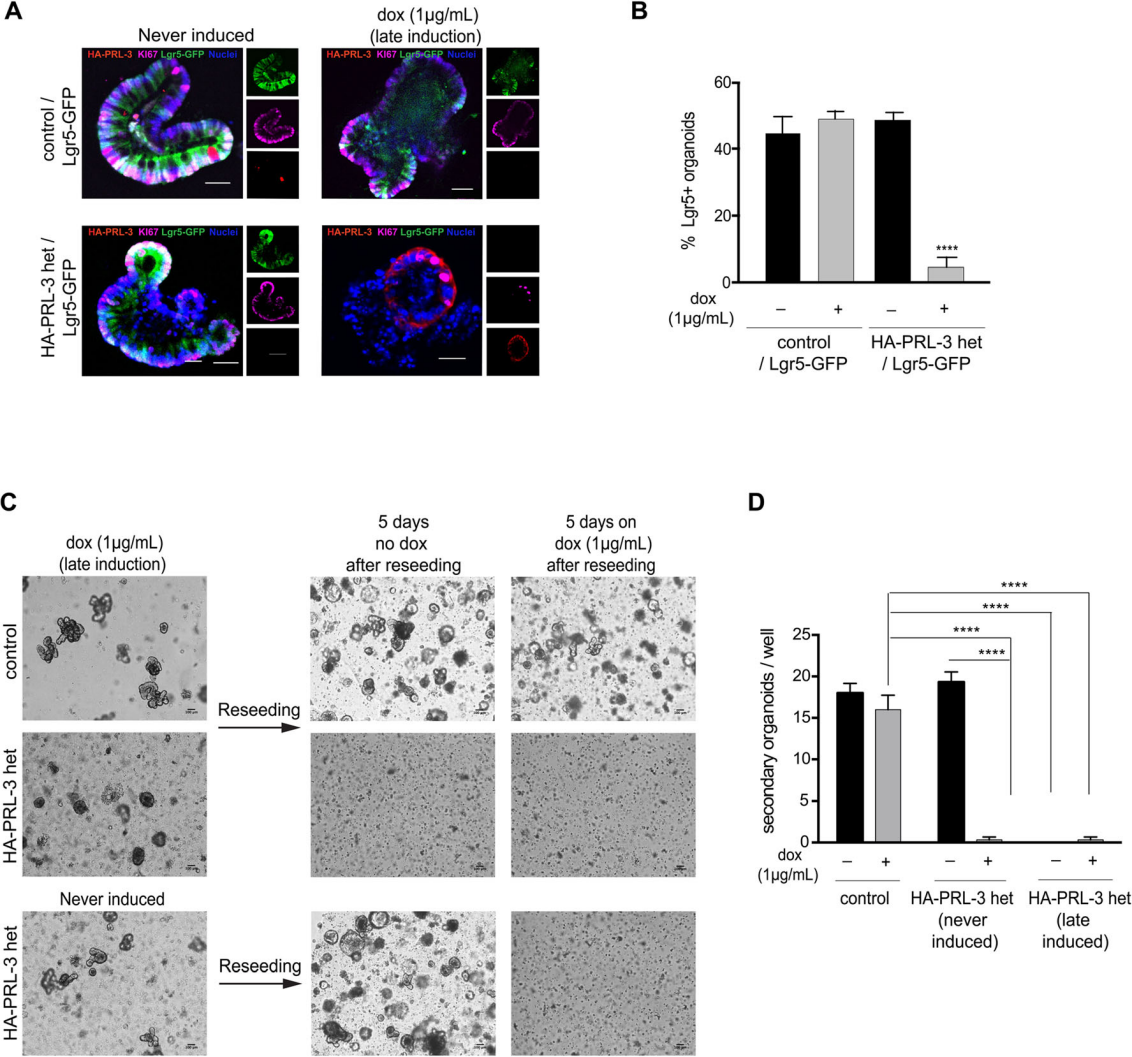
Overexpression of PRL-3 leads to the death of ISCs. **a** Representative confocal microscopy images of IF in small intestinal organoids derived from HA-PRL-3 wt/Lgr5-GFP (control) or HA-PRL-3 het/Lgr5-GFP mice never induced or late induced with dox. IF with HA (red) and KI67 proliferation marker (magenta) antibodies. GFP (green) native marks the Lgr5 positive cells. Scale bar: 100 μm. **b** Percentage of Lgr5-GFP positive organoids observed in (**a**). **c** Representative bright field inverted microscopy images of the small intestinal organoids derived from control or HA-PRL-3 het mice late induced or without dox (never induced). After 4 days on dox the cultures were split and reseeded in new fresh Matrigel, then treated or not with 1 μg/mL dox for 5 more days. Scale bars: 100μm (**d**) Representation of the number of secondary organoids per well, obtained after the reseeding. On the x-axis the genotypes for the primary derived organoids are represented and +/- corresponds to the dox treatment of the secondary organoids. **b**, **d** Data are mean ± s.d. N=3, **P≤0.01, ****P≤0.0001, ordinary two-tailed two-way ANOVA with Tukey’s multiple comparison test

To further verify that PRL-3 overexpression triggers the death of the ISCs selectively, crypts from HA-PRL-3/Lgr5-GFP mice were isolated and cultured in 3D for 2 days without the induction of PRL-3 expression leading to fully developed organoids. Then, HA-PRL-3 overexpression was induced for the next 2 days (late induction). These organoids showed the same round-shaped phenotype that was previously observed for derived intestinal organoids of the bi-transgenic mice (Fig. 3). Indeed, a strong decrease in Lgr5+ cells was observed (Fig. 4a, b), confirming that PRL-3 overexpression is also toxic for the ISCs in vitro. Moreover, the decrease observed in Lgr5+ cells correlates with a qualitative decrease of proliferative (KI67+) cells (Fig. 4a), which can explain the failure in organoid development after PRL-3 expression. This very strong and fast effect in vitro suggests that like in HA-PRL-3 homo mice in vivo, Paneth cells were not able to compensate for the reduction of ISCs.

Since some proliferative cells were detected in all organoids expressing PRL-3 (Fig. 4a and Fig S4B), we wondered whether any organoid could escape from the PRL-3-dependent toxicity, surviving and still being able to proliferate. To address this question, first a dox wash out experiment was carried out. Organoids derived from bi-transgenic mice (HA-PRL-3 het/R26-rtTA het) were grown for 2 days, and then PRL-3 overexpression was induced for 2 more days (late induction) by the addition of dox. As control the same organoids without dox treatment were used (never induced). The organoids expressing PRL-3 showed the round-shaped phenotype previously observed. Then these organoids were grown in dox-free media for 2 more days. Although dox was removed from the media, no rescue was observed (Fig S4 C, D), and the organoids showed the round-shaped phenotype confirming that the ISCs in the PRL-3 overexpressing organoids were compromised and had lost the ability to develop again the crypt-like domain. Second, a reseeding experiment was carried out. Crypts derived from HA-PRL-3 wt/R26-rtTA het, control, or HA-PRL-3 het/R26-rtTA het organoids treated or not with dox were trypsinized to single cells and seeded again in 3D (second seeding). Contrary to the control, the organoids derived from the PRL-3 overexpressing aberrant round-shaped organoids were not able to build new 3D structures in the second seeding, not even in the absence of dox (Fig. 4c, d), demonstrating that the primary organoids have lost their stemness capacity.

## Discussion

Expression and action of PRL-3 phosphatase are associated with malignant behavior in tumor progression and metastasis. Here, we generated an inducible PRL-3 transgenic mouse model to conditionally overexpress human PRL-3 to mimic conditions observed in patients as close as possible. This new model has the advantage of introducing one single copy of the transgene in the colA1 locus, avoiding random integration [35]. Although PRL-3 mRNA was found in all the tissues tested, PRL-3 protein expression was only detected in a subset of tissues, confirming previous results observed for the pTET-on-pTRE2-Ptp4a3 TG mice [12]. This phenomenon is likely due to the post-transcriptional regulation of PRL-3 protein, for which a number of studies have described possible mechanisms [39].

PRL-3 heterozygous overexpression was not sufficient to induce tumor development in mice even after 12 months of induction. However, we show that its expression reduces the population of Lgr5+ ISCs. This phenomenon has been previously associated with a more rapid fixation of Apc-defective ISCs within the intestinal crypt leading to an accelerated tumorigenesis [28]. Therefore, these observations suggest that PRL-3 could facilitate tumor initiation and development in the long run, upon additional—possibly environmental—hits. Accordingly, PRL-3 KO mice treated with AOM combined with DSS exhibited less tumors compared to wt mice [11], and Lian et al. showed that PRL-3 overexpressing TG mice developed more tumors compared to wt mice under DSS treatment [12]. Since DSS is a pro-inflammatory treatment, it can be presumed that PRL-3 could be involved in inflammation-related colon malignancy. Consistently, we found that the amount of Paneth cells increases in intestinal crypts upon PRL-3 overexpression, which has been associated with inflammatory bowel disease developed after intestinal injury [29–33]. These data together suggest that PRL-3 expression, by itself, is not sufficient to develop tumors in this system but that it primes the intestinal tissue for cancer initiation.

When comparing HA-PRL-3 heterozygous mice (on dox) to control mice, no difference in body weight was observed. However, in contrast, HA-PRL-3 homozygous mice showed a dramatic reduction in body weight after 2 weeks of dox treatment together with a short lifespan, indicative of a heavily compromised gut function, which we observed in histological and IF staining as well as by flow cytometric quantification of the crypt composition (Fig. 2a–d). These observations are likely due to the ablation of the ISCs after 7 days of induction in HA-PRL-3 homozygous mice compared to a milder reduction of the population of these Lgr5+ cells observed in the heterozygous mice. Nevertheless, although our data points toward the early death of homozygous mice being due to the compromised gut function, we cannot exclude that other PRL-3-induced organ defects could contribute to the early death. Taken together, these results suggest a decrease of Lgr5+ ISCs in a PRL-3 dose-dependent manner.

The decrease in Lgr5+ ISCs observed in the present study correlated with an increase in Paneth cells counts, in line with previous reports where the damage of the ISCs led to the expansion of Paneth cell numbers [40]. This Paneth cell increase could be explained by their role in niche signaling maintenance [14, 40]. In fact, Paneth cells also have been suggested to maintain the stemness capacity of intestinal tissue when ISCs were reduced [14, 41]. Nevertheless, the dramatic loss of Lgr5+ cells in HA-PRL-3 homo mice may be too fast to differentiate in order to increase the Paneth cell population as much as in the HA-PRL-3 het mice. Therefore, these mice would not have enough Paneth cells to support the lack of Lgr5+ ISCs explaining their early death. In line with this hypothesis, a decrease in KI67+ proliferative cells was also detected in homozygous mice compared to control or heterozygous mice already after 2 days on dox. Thus, the lack of cell proliferation and, as a consequence, disruption of intestine cell homeostasis can explain the early death of the homozygous mice.

The sensitivity of Lgr5+ ISCs to PRL-3 overexpression was also confirmed in vitro. Crypts derived from HA-PRL-3 het/R26-rtTA het mice failed to form organoids at early induction and lacked the crypt domain at late induction of PRL-3 expression. This strong phenotype prevented us from studying differences between the organoids derived from the homozygous and heterozygous PRL-3 expressing mice, as the former showed the same phenotype. The complete depletion of ISCs in the PRL-3 heterozygous organoids was much stronger than the mild reduction of Lgr5+ ISC counts observed in vivo. This discrepancy could be due to a more complex in vivo intestine cell homeostasis regulation that could counterbalance the toxic effect of PRL-3 on the intestinal crypts [24, 25].

Mechanistically, PRL-3 is known for its pleiotropic effects [1, 10, 12], and therefore it is highly challenging to elucidate which particular PRL-3-mediated processes cause ISC toxicity. In addition, the severe impact of PRL-3 expression on organoid formation and the resulting very short life of these cultures makes the use of intestinal organoids to study the molecular mechanisms of PRL-3 technically not feasible. Thus, future in vivo studies will have to shed light on the mechanisms involved.

In summary, we provide here insights into the cellular consequences of PRL-3 expression in intestinal tissue in vivo. Our data indicate that PRL-3 expression limits the self-renewal capacity of the intestine by leading to the death of ISCs. This reduction can in turn lessen the ability of the intestine to heal itself, making it more prone to inflammation and cancer initiation [28–33], thus suggesting PRL-3 as a new therapeutic opportunity for cancer prevention by inhibiting it at the stage of (chronic) inflammation.

## Methods

### Plasmids

Human PRL-3 cDNA was amplified with the following primers including HA in the forward and EcoRI restriction site in both primers: Forward: CGCCGAATTCCACCATGGCTTACCCATACGATGTTCCAGATTACGCTGCTCGGATGAACCGCCCGGCCCCGGTGG and reverse: GCGTGAATTCCTACATAACGCAGCACCGGGTCTTGTG

HA-PRL-3 was cloned with EcoRI into the pgk-ATG-frt-SAdpA-tetO vector (pBS31).

### Generation of HA-PRL-3/R26-rtTA transgenic mice

Fifty micrograms of a plasmid containing the HA-*PRL-3* human cDNA under the control of the doxycycline (dox) responsive promoter (tetO) (see plasmids) was inserted downstream of the Col1A1 locus (Fig s1A) by co-electroporation with 25 μg of a Flpe recombinase containing vector into KH2-ES cells [35]. After ES cell electroporation, three positive clones were able to grow in presence of 150 μg/mL hygromycin. These clones (A1, B1, and B2) were expanded, and HA-PRL-3 protein expression was checked by western blot after dox treatment with anti HA mouse primary antibody (Roche) (Fig s1B and Sup1). Subsequently, clones A1 and B1 were independently microinjected into blastocysts fertilized C57BL/6J embryos. Thus, two bi-transgenic (HA-PRL-3 het/R26-rtTA het) mice from these two independent litters were used to expand the two TG mouse colonies separately, corresponding to founders A1 and B1. In order to discard possible random transgenesis, the phenotype of each mouse colony was independently analyzed, and the same phenotype was observed in both.

### Animal husbandry and genotyping

HA-PRL-3/R26-rtTA and Lgr5-EGFP-IRES-CreERT2 [21] mice were kept in pathogen-free housing at EMBL-Rome and EMBL-Heidelberg, with ethical approval from the corresponding Animal Welfare and Ethical Review Bodies and national and European legislations. Mice with different combinations of the *HA-PRL-3* and *R26-rtTA* alleles were fed with 625 ppm doxycycline-impregnated food in order to achieve ubiquitous transgene expression and evaluate HA-PRL-3 expression tolerance. Tail DNA for genotyping was obtained via incubation in 200 μL 0.05M NaOH at 98 °C for 1.5 h and subsequent neutralization with 20 μL 1M Tris HCl pH 7.5.

Mice were genotyped using DreamTaq DNA polymerase from Thermo Fisher (EP0701) with the following primers (5′-3′) and PCR programs:

- R26-rtTA: AAAGTCGCTCTGAGTTGTTAT; GGAGCGGGAGAAATGGATATG and GCGAAGAGTTTGTCCTCAACC and PCR program: 95°C × 5 min; [94°C× 30 s; 60°C× 1 min; 72°C ×1.5 min] × 28 cycles; 72^o^C × 5min
- HA-PRL-3: GCACAGCATTGCGGACATGC; CCCTCCATGTGTGACCAAGG and GCAGAAGCGCGGCCGTCTGG and PCR program: 94°C × 2 min; [94°C× 15 s; 60°C× 30 s; 72°C ×30 s] × 29 cycles; 72°C × 1min
- Lgr5-GFP: CACTGCATTCTAGTTGTGG and CGGTGCCCGCAGCGAG and PCR program: 95°C × 1 min; [95°C× 15 s; 64°C× 15 s; 72°C ×1.5 min] × 2 cycles; [95°C× 15 s; 61°C× 15 s; 72°C ×1.5 min] × 2 cycles; [95°C× 15 s; 58°C× 15 s; 72°C ×1.5 min] × 23 cycles; [95°C× 15 s; 55°C× 15 s; 72°C ×1.5 min] × 18 cycles; 72°C × 10 min

### RNA analysis

For RNA analysis, snap frozen tissues were homogenized using mortar and pestle while maintaining temperature at −80 °C using dry ice. RNA extraction was carried out following manufacturer’s recommendations (RNeasy Mini Kit, Qiagen). RNA was treated with DNaseI (Roche) to eliminate any contaminating DNA. PRL-3 mRNA level was determined by performing one-step reverse transcription-PCR (Qiagen) according to the manufacturer’s instructions and using the primers (5′-3′): forward (GATTACGCTGCTCGGATGAAC) annealing in HA and reverse (TTCCACTACCTTGCCGGGC) annealing in PRL-3 sequence; and the PCR program: 95°C × 2 min; [95°C× 30 s; 58°C× 45 s; 72°C ×45 s] × 30 cycles; 72°C × 5min.

### Protein analysis

For protein extraction and immunoblots, mouse tissues or organoids were lysed on ice in RIPA lysis buffer (50mM Tris HCl pH 7,4, 150 mM NaCl, 1% NP40, 0,25% sodium deoxycholate) containing complete protease inhibitors (Roche). For organoids beforehand, lysis Matrigel was removed using cell recovery solution (Corning) as described in manufacturer’s protocol. Samples were then boiled for 10 min and cleared by centrifugation. Equal amounts of lysate protein were separated performing SDS-PAGE. Western blotting was performed with anti HA mouse primary antibody (Roche) and anti actin rabbit primary antibody (SIGMA) and then with secondary anti-mouse or anti-rabbit antibodies conjugated to horseradish peroxidase (Sigma and Amersham Biosciences, respectively). Visualization was achieved with the Western Lightning Plus-ECL solution (PerkinElmer).

### Immunohistochemistry

For immunohistochemical staining, formalin-fixed paraffin embedded 5-μm sections derived from different mouse tissues were used. Following deparaffinization with xylene and rehydration with graded ethanol, antigen retrieval was performed using 0.09% (v/v) unmasking solution (Vector Labs) for 30 min in a steamer. Inactivation of endogenous peroxidases was carried out using 3% hydrogen peroxide (Sigma) for 10 min. Primary antibodies used, anti HA mouse (Covance clone 11; MMs-101R), anti ki67 pre-diluted (MEDAC 275R-18) mouse, and/or anti cleaved caspase-3 rabbit (cell signaling 9664S) were diluted in 10% goat serum (Jackson Immuno) in TBS-Tween 0.1%. Secondary antibody staining and biotin-streptavidin incubation were performed using species-specific VECTASTAIN Elite ABC kits (Vector Labs). DAB Peroxidase Substrate kit (Vector Labs) was utilized for antibody detection. Eosin Y and hematoxylin were from Bio-Optica and Vector Labs. Tissue pictures were obtained using Tissue tek. Pictures were then analyzed using the QuPath software from https://qupath.github.io/.

### Immunofluorescence

For the immunofluorescence staining of 3D cell cultures, first the samples were rinsed with phosphate-buffered saline (PBS) twice and fixed with 4% paraformaldehyde (PFA) for 20 min. PFA was then quenched with 0.1 M glycine in PBS for 10 min, and then cells were permeabilized with 0.5% Triton X-100 in PBS for 10 min and blocked with 10% FBS in PBS with 0.5% Triton X-100 (blocking solution) for 60 min. Then, samples were incubated with primary antibodies anti HA rat high affinity (Roche 11867423001), and cleaved caspase-3 rabbit (cell signaling 9664S), or anti KI67 pre-diluted (MEDAC 275R-18) mouse in blocking solution at 4°C overnight, followed by washing and incubation with the corresponding secondary Alexa-fluorophore-conjugated antibody (1/1000, Invitrogen) for 1 h. Rhodamine-conjugated phalloidin 1:1000 (R415, Invitrogen) and Hoechst 33342 (10 μg/ml) were also used for membrane and nuclei labeling, respectively. Cell images were acquired at room temperature on a spinning disc confocal microscope PerkinElmer UltraView ERS FIJI software (National Institutes of Health). Contrast or brightness adjustment was applied to the whole image. In FIJI software, a 50-pixel rolling ball radius was used to subtract background, and a 1-pixel-radius median filter was applied.

For immunofluorescence in intestinal frozen tissue samples OCT (Tissue Tek) embedded 10-μm tissue sections were warmed for 30 min and then washed with PBS-Tween 1% for 15 min. The sections were then blocked with 2% BSA (SIGMA) and 8% goat serum (Jackson Immuno-research) in PBS-Tween 0.1% for 1h. Primary antibodies in the blocking buffer were added to the slides and incubated overnight at 4°C (HA rat Roche 11867423001 (1:1000) and rabbit anti-GFP Abcam 6556 (1:1000)). Samples were washed with PBS. The secondary antibodies were also dissolved in blocking buffer (Goat anti-Rat IgG (H+L) Alexa Fluor 647 A-21247; Goat anti-rabbit IgG (H+L) Alexa Fluor 594 A11037; Hoechst 33342 H3570 (1:1000, respectively) Samples were washed with PBS and mounted with Fluoromount-G (eBioScience). Immunofluorescence images were acquired at room temperature on a Zeiss LSM880 laser scanning confocal microscope using a Plan-Apochromat 25×0.8 NA glycerin objective. Images were processed using FIJI software. Contrast or brightness adjustment was applied. For visualization reasons, the brightness of the red HA-PRL-3 signal in the HA-PRL-3 homo/Lgr5-GFP sample was slightly increased compared to the HA-PRL-3 het/Lgr5-GFP sample (Fig. 2c). In FIJI software, a 50-pixel rolling ball radius was used to subtract background, and a 1-pixel-radius median filter was applied.

### Three-dimensional organotypic assays

As previously reported [37] and briefly summarized here, SIs or colons were removed, washed with cold PBS not containing magnesium chloride and calcium (PBS−/−) opened longitudinally, and then cut into 3–5-mm fragments. Pieces were washed several times with cold PBS−/− until clean, washed 2–3 with PBS−/− EDTA (10 mM), and incubated on ice for 90 min, gently shaking. Crypts were then mechanically separated from the connective tissue by more rigorous shaking and then filtered through a 70-μm mesh into a 50-mL conical tube to remove villus material and tissue fragments. Isolated crypts were washed twice with DMEM F12 (Life Technologies 21331-046), 2mM Glutamax (Life Technologies 35050-061), 10 mM Hepes, and 5mL penicillin/streptomycin and centrifuged at 1100rpm × 4min at 4°C. The crypts were then embedded in 100% Matrigel (Corning 356231 growth factor reduced) at 5–10 crypts per μL and cultured in the IntestiCult Organoid Grow Media for mouse (Stem Cell Technologies) or used for FC experiments as described below. For the *analog 3* experiment, a precoating with 100% Matrigel was carried out, and the intestinal cells were embedded in 40% Matrigel instead of 100% to ensure that the inhibitor can reach the organoids.

The intestinal organoids were grown in the presence of doxycycline (SIGMA) at the indicated concentration and time for each experiment and/or in presence of the PRL inhibitor *A3* (Enamine) [38] or in DMSO as a control.

For reseeding, primary organoids in a 24 well plate were treated with 2μL collagenase (Worthington Biochemical corp.) and 2μL liberase TM (Roche) for 2h at 37°C. Then the gel was completely disaggregated mechanically with a p1000 pipette and collected in a 15-mL tube with warm PBS -/- followed by 300g × 5-min centrifugation. For single cells, the pellet containing rest of the gel and the organoids was washed with warm PBS-/- and then incubated with 0.5% trypsin (Thermo Fisher) at 37°C for 5 min. The trypsin reaction was stopped with DMEM F12 including 2mM Glutamax, 10 mM Hepes, 5mL penicillin/streptomycin, and 10% fetal bovine tetracycline free Serum (Peqlab). Cell pellet was collected by centrifugation at 300g × 5 min. The cells were then resuspended in 1mL of warm IntestiCult media and seed at 5–10 crypts per μL density (50 μL per well in a 24 well plate) and embedded in Matrigel as described above.

### Viability assay in organoids

Mouse organoid cultures were set as above described. After 4 days of culture, single cells were obtained as described above for reseeding cultures. The trypsin reaction was stopped with 15 mL DMEM F12 including 2mM Glutamax, 10 mM Hepes, 5mL penicillin/streptomycin and 10% fetal bovine tetracycline free serum, and 7 μL DNaseI (Roche) and incubated at RT for 7 min. Cells were recuperated by centrifugation at 300 g × 5 min and resuspended in 200 μL of PBS-/-. Then the cells were passed through a 70-μm cell strainer, and 0.5 μg/mL of propidium Iodide (SIGMA) was added. PI signal was measured in a BD LSR Fortessa cell analyzer.

### Flow cytometry

For ISC isolation, the crypt suspensions were isolated from the intestine tissue and dissociated to individual cells following the described method above for 3D organotypic assays. The crypt cell pellet was then washed with cold PBS−/− and centrifuge at 1100 r.p.m 4 min. Then the cells were incubated with 300 μL of an antibody cocktail comprising anti mouse CD45-PerCP55 (BD) 1/400, CD31-perCP55 (BD) 1/400 and Ter119- perCP55 (BD), 1/400 CD24-BV421 (BD) 1/200, CD117-PECy7 (biolegend) 1/800, and Epcam-PE (BD) 1/200.

After antibody incubation, pellet was washed again with PBS−/− and resuspended in 300 μL of PBS−/−. Dead cells were excluded from the analysis with the viability dye 7-AAD (Thermo Scientific). The different cell populations conforming the intestinal crypts derived from KH2-HA-PRL-3/R26 rtTA × Lgr5-EGFP-IRES-CreERT2 were sorted [52,53] as (a) ISCs as Lgr5- EGFP^hi^ Epcam^+^CD24^low/−^CD31^−^Ter119^−^CD45^−^7-AAD^−^, (b) progenitor cells as EGFP^low^Epcam^+^CD24^low/−^CD31^−^Ter119^−^CD45^−^7-AAD , and (c) Paneth cells as CD24^hi^Sidescatter^hi^Lgr5- EGFP^−^Epcam^+^CD31^−^Ter119^−^CD45^−^7-AAD^−^ and with a BD FACS Aria TM Fusion sorter and analyzed with FACSDiva 8.0.1 software.

### Statistical analysis

Ordinary two-tailed one- or two-way ANOVA and Tukey’s or Dunnett’s multiple comparison tests were performed to study statistical significance: *P≤0.05, **P≤0.01, ***P≤0.001, and ****P≤0.0001. Statistical analysis and graphs were generated with Prism (GraphPad).

Control samples for in vivo experiments were obtained from animals containing the transgenes but kept on a normal diet or HA-PRL-3 wt/R26-rtTA het mice on dox.

Non-induced organoid cultures (without dox) derived from HA-PRL-3 wt/R26-rtTA het mouse intestine on a normal diet were considered as controls in the in vitro assays.

## **Supplementary information**

Fig s1. **Design and characterization of PRL-3 TG mice. A:** Schematic representation of the plasmid construct encoding the HA-*Ptp4a3* gene used for ES cell electroporation and of the strategy for insertion in the *ColA1* locus. **B:** Western blot analysis of ES clones electroporated with HA-PRL-3 vector using HA antibody. Cells were treated in vitro with 1μg/mL of dox for 48h before the protein extraction. **C:** PRL-3 mRNA extracted from the denoted organs from HA-PRL-3 het / R26-rtTA het TG mice (FA1) after 15 days on dox was assayed using one-step RT-PCR using mRNA encoding β-actin as a loading control. **D:** Western blots using tissue samples derived from HA-PRL-3 wt / R26-rtTA het (control) fifteen days on dox and HA-PRL-3 het / R26-rtTA het (HA-PRL-3 het) mice fed with dox impregnated pellets for 0 (not treated), 2 or 15 days. HA and actin (loading control) signals were detected using specific antibodies. **E**: spleen weight or **F:** liver weight relative to body weight (a.u. = arbitrary units), in control and HA-PRL-3 het mice from the FA1 colony treated 12 months with dox. N=10. **G:** Body weight by gender of males (N=4) and females (N=8) from the FB1 mouse colony fed with dox food for 12 months showed no significant (ns) differences in body weight (g=grams) by Oneway Anova with Turkey’ s multiple comparison test compared to control mice. **H, I** same representations as in **E, F** but for FB1 mouse colony. N=11. **D, E, G, H**: Unpaired T test, non-significant differences. **K**: Kaplan Meier survival curve of HA-PRL-3 het / R26-rtTA het (PRL-3 het), HA-PRL-3 homo / R26-rtTA het (PRL-3 homo) and HA-PRL-3 wt / R26-rtTA het (control) mice without doxycycline. N=10 for each genotype. All genotypes presented the same life span when PRL-3 expression was not induced with doxycycline. (PNG 1038 kb)

High Resolution Image (TIF 1259 kb)

Fig S2. A: Western Blot analysis of small intestine derived tissue samples from HA-PRL-3 wt / R26-rtTA het (control), HA-PRL-3 het/R26-rtTA het (HA-PRL-3 het) and HA-PRL-3 homo / R26-rtTA het (HA-PRL-3 homo) mice treated two days with dox. HA signal was detected using specific antibodies. β actin was used as a loading control. **B:** Quantification of the Western Blot shown in A. PRL-3 HA expression is presented in relation to β actin loading control (HA-PRL-3 / β actin ratio). HA-PRL-3 het and HA-PRL-3 homo demonstrate the average of sample 1 and 2, respectively. **C**: Representative images of KI67 staining of colon biopsies from mice treated 3 months with dox upper panel, or 12 months middle panel. The lower panel shows the HA staining for PRL-3 detection in mice after 12 months on dox food. **D:** Percentage of KI67 positive cells (relative to total cells counted) in the colonic crypts in the different conditions represented in **C**. 1000 cells were analyzed per tissue sample. Values represent mean ± s.d. of three biological replicates. Statistics: *P*≤0.1 *t*-test, compared to control mice. (PNG 2868 kb)

High Resolution Image (TIF 3616 kb)

Fig S3. **Phenotype and HA-PRL-3 expression of colon (A, C, E) and SI (B, D, F) organoids.** 3D cell cultures derived from HA-PRL-3 het / R26-rtTA het mice and HA-PRL-3 homo / R26-rtTA het mice were grown for 2 days after passaging. Subsequently, HA-PRL-3 expression was induced in vitro with 1μg/mL of dox for 2 days (late induction). **A, B:** Representative bright field inverted microscopy images. Pictures were taken after four days in culture for all conditions. All dox-induced organoids showed the death phenotype compared to non-induced cultures. Scale bar = 50 μm. **C, D:** Western blot analysis of 3D cell culture after four days. HA signal was detected using specific antibodies. β-actin was used as a loading control. **E, F:** Quantification of the Western blot shown in C, D. PRL-3 HA expression is presented in relation to β actin loading control (HA-PRL-3 / β actin ratio) and normalized to HA-PRL-3 het induced with dox signal. HA expression in homozygous cultures were increased compared to heterozygous. (PNG 1652 kb)

High Resolution Image (TIF 2102 kb)

Fig S4. **A**: PI assay to detect cell death in organoids never induced or late induced. **B:** Percentage of KI67 positive organoids observed in Fig 4a**.** The quantification corresponds to the percentage of organoids with any KI67 positive cell with no differences observed between control and PRL-3 het organoids. Although, the pictures in A shows higher number of KI67 positive cells in the control compare to HA-PRL-3 het organoids (with only one or two cells stained for KI67), the number of KI67 positive cells in each organoid was not possible to quantify due to the 3D structure of the organoids. **C:** Representative bright field inverted microscopy images of small intestinal organoids derived from HA-PRL-3 het mice never induced (no dox) or late induced (dox added in the growth media after 2 days in culture). After 2 days of induction the dox was removed from the media and the organoids were grown in dox free media for two more days. Scale bars: 100 μm. **D:** Percentage of organoids developed with branches, relative to total organoids, before and after DOX withdrawal. Data are mean ± s.d. N=3, ns: not significant, ****P≤0.0001 ordinary two-tailed one-way ANOVA with Tukey's multiple comparison test. (PNG 2313 kb)

High Resolution Image (TIF 2832 kb)

ESM 5 **Entire Western blot membranes. A:** Entire western blot membranes for the ES clones electroporated with HA-PRL-3 vector using HA antibody and showed in Fig S1B. **B:** Entire western blot membranes showed in Fig 1a and Fig S1D using tissue samples derived from HA-PRL-3 wt / R26-rtTA het (control) 15 days on dox and HA-PRL-3 het / R26-rtTA het (HA-PRL-3 het) mice fed with dox impregnated pellets for 0 (not treated), 2 or 15 days. Upper membranes correspond to anti HA antibody for HA-PRL-3 detection and lower membranes anti β actin antibody as a loading control. **C:** Entire western blot membranes showed in Fig S2A using small intestine tissue samples derived from HA-PRL-3 wt / R26-rtTA het (control), HA-PRL-3 het/R26-rtTA het (HA-PRL-3 het) and HA-PRL-3 homo / R26-rtTA het (HA-PRL-3 homo) 2 days on dox. Upper membranes correspond to anti HA antibody for HA-PRL-3 detection and lower membranes correspond to β actin used as a loading control. **D:** Entire western blot membranes showed in Fig S3C,D. Upper membranes correspond to anti HA antibody for HA-PRL-3 detection and lower membranes anti β actin antibody as a loading control. For further information see Fig S3 caption. (PDF 2433 kb)

## References

[1] Rios P, Li X, Köhn M (2013). Molecular mechanisms of the PRL phosphatases. FEBS J.

[2] Guzinska-Ustymowicz K, Pryczynicz A (2011). PRL-3, An emerging marker of carcinogenesis, is strongly associated with poor prognosis. Anti Cancer Agents Med Chem.

[3] Saha S, Bardelli A, Buckhaults P, Velculescu VE, Rago C, St Croix B, Romans KE, Choti M, Lengauer C, Kinzler KW, Vogelstein B (2001). A phosphatase associated with metastasis of colorectal cancer. Science.

[4] Thura M, Al-Aidaroos AQ, Gupta A, Chee CE, Lee SC, Hui KM, Li J, Guan YK, Yong WP, So J, Chng WJ, Ng CH, Zhou J, Wang LZ, Yuen JSP, Ho HSS, Yi SM, Chiong E, Choo SP, Ngeow J, Ng MCH, Chua C, Yeo ESA, Tan IBH, Sng JXE, Tan NYZ, Thiery JP, Goh BC, Zeng Q (2019). PRL3-zumab as an immunotherapy to inhibit tumors expressing PRL3 oncoprotein. Nat Commun.

[5] Qian F, Li YP, Sheng X, Zhang ZC, Song R, Dong W, Cao SX, Hua ZC, Xu Q (2007). PRL-3 siRNA inhibits the metastasis of B16-BL6 mouse melanoma cells in vitro and in vivo. Mol Med.

[6] Al-Aidaroos AQ, Zeng Q (2010). PRL-3 phosphatase and cancer metastasis. J Cell Biochem.

[7] Radke I, Götte M, Smollich M, Scharle N, Kiesel L, Wülfing P (2017). Expression of PRL-3 regulates proliferation and invasion of breast cancer cells in vitro. Arch Gynecol Obstet.

[8] Bessette DC, Qiu D, Pallen CJ (2008). PRL PTPs: Mediators and markers of cancer progression. Cancer Metastasis Rev.

[9] Luján P, Varsano G, Rubio T, Hennrich ML, Sachsenheimer T, Gálvez-Santisteban M, Martín-Belmonte F, Gavin AC, Brügger B, Köhn M (2016). PRL-3 disrupts epithelial architecture by altering the post-mitotic midbody position. J Cell Sci.

[10] Hardy S, Kostantin E, Hatzihristidis T, Zolotarov Y, Uetani N, Tremblay ML (2018). Physiological and oncogenic roles of the PRL phosphatases. FEBS J.

[11] Zimmerman MW, Homanics GE, Lazo JS (2013). Targeted deletion of the metastasis-associated phosphatase Ptp4a3 (PRL-3) suppresses murine colon cancer. PLoS One.

[12] Lian S, Meng L, Yang Y, Ma T, Xing X, Feng Q, Song Q, Liu C, Tian Z, Qu L, Shou C (2017). PRL-3 promotes telomere deprotection and chromosomal instability. Nucleic Acids Res.

[13] Van Der Heijden M, Vermeulen L (2019). Stem cells in homeostasis and cancer of the gut. Mol Cancer.

[14] Clevers HC, Bevins CL (2013). Paneth cells: maestros of the small intestinal crypts. Annu Rev Physiol.

[15] Kuhnert F, Davis CR, Wang HT, Chu P, Lee M, Yuan J, Nusse R, Kuo CJ (2004). Essential requirement for Wnt signaling in proliferation of adult small intestine and colon revealed by adenoviral expression of Dickkopf-1. Proc Natl Acad Sci.

[16] Korinek V, Barker N, Willert K, Molenaar M, Roose J, Wagenaar G, Markman M, Lamers W, Destree O, Clevers H (1998). Two members of the Tcf family implicated in Wnt/β-catenin signaling during embryogenesis in the mouse. Mol Cell Biol.

[17] Pinto D, Clevers H (2005). Wnt control of stem cells and differentiation in the intestinal epithelium. Exp Cell Res.

[18] van der Flier LG, Clevers H (2009). Stem cells, self-renewal, and differentiation in the intestinal epithelium. Annu Rev Physiol.

[19] de Lau W, Barker N, Low TY, Koo BK, Li VS, Teunissen H, Kujala P, Haegebarth A, Peters PJ, van de Wetering M, Stange DE, van Es JE, Guardavaccaro D, Schasfoort RB, Mohri Y, Nishimori K, Mohammed S, Heck AJ, Clevers H (2011). Lgr5 homologues associate with Wnt receptors and mediate R-spondin signalling. Nature.

[20] Carmon KS, Gong X, Lin Q, Thomas A, Liu Q (2011). R-spondins function as ligands of the orphan receptors LGR4 and LGR5 to regulate Wnt/-catenin signaling. Proc Natl Acad Sci.

[21] Barker N, van Es JH, Kuipers J, Kujala P, van den Born M, Cozijnsen M, Haegebarth A, Korving J, Begthel H, Peters PJ, Clevers H (2007). Identification of stem cells in small intestine and colon by marker gene Lgr5. Nature.

[22] van Es JH, Jay P, Gregorieff A, van Gijn ME, Jonkheer S, Hatzis P, Thiele A, van den Born M, Begthel H, Brabletz T, Taketo MM, Clevers H (2005). Wnt signalling induces maturation of Paneth cells in intestinal crypts. Nat Cell Biol.

[23] Farin HF, Van Es JH, Clevers H (2012). Redundant sources of Wnt regulate intestinal stem cells and promote formation of Paneth cells. Gastroenterology.

[24] Tian H, Biehs B, Warming S, Leong KG, Rangell L, Klein OD, de Sauvage FJ (2011). A reserve stem cell population in small intestine renders Lgr5-positive cells dispensable. Nature.

[25] Metcalfe C, Kljavin NM, Ybarra R, De Sauvage FJ (2014). Lgr5+ stem cells are indispensable for radiation-induced intestinal regeneration. Cell Stem Cell.

[26] Snippert HJ, van der Flier LG, Sato T, van Es JH, van den Born M, Kroon-Veenboer C, Barker N, Klein AM, van Rheenen J, Simons BD, Clevers H (2010). Intestinal crypt homeostasis results from neutral competition between symmetrically dividing Lgr5 stem cells. Cell.

[27] Lopez-Garcia C, Klein AM, Simons BD, Winton DJ (2010). Intestinal stem cell replacement follows a pattern of neutral drift. Science.

[28] Huels DJ, Bruens L, Hodder MC, Cammareri P, Campbell AD, Ridgway RA, Gay DM, Solar-Abboud M, Faller WJ, Nixon C, Zeiger LB, McLaughlin ME, Morrissey E, Winton DJ, Snippert HJ, van Rheenen J, Sansom OJ (2018). Wnt ligands influence tumour initiation by controlling the number of intestinal stem cells. Nat Commun.

[29] King SL, Mohiuddin JJ, Dekaney CM (2013). Paneth cells expand from newly created and preexisting cells during repair after doxorubicin-induced damage. AJP Gastrointest Liver Physiol.

[30] Tanaka M, Riddell RH, Saito H, Soma Y, Hidaka H, Kudo H (1999). Morphologic criteria applicable to biopsy specimens for effective distinction of inflammatory bowel disease from other forms of colitis and of Crohn’s disease from ulcerative colitis. Scand J Gastroenterol.

[31] Verburg M, Renes IB, Meijer HP, Taminiau JA, Büller HA, Einerhand AW, Dekker J (2000). Selective sparing of goblet cells and Paneth cells in the intestine of methotrexate-treated rats. Am J Physiol Liver Physiol.

[32] Wong WM, Stamp GW, Elia G, Poulsom R, Wright NA (2000). Proliferative populations in intestinal metaplasia: evidence of deregulation in Paneth and goblet cells, but not endocrine cells. J Pathol.

[33] Helmrath MA, Fong JJ, Dekaney CM, Henning SJ (2006). Rapid expansion of intestinal secretory lineages following a massive small bowel resection in mice. AJP Gastrointest Liver Physiol.

[34] Dekaney CM, Gulati AS, Garrison AP, Helmrath MA, Henning SJ (2009). Regeneration of intestinal stem/progenitor cells following doxorubicin treatment of mice. AJP Gastrointest Liver Physiol.

[35] Beard C, Hochedlinger K, Plath K, Wutz A, Jaenisch R (2006). Efficient method to generate single-copy transgenic mice by site-specific integration in embryonic stem cells. Genesis.

[36] Sato T, Vries RG, Snippert HJ, van de Wetering M, Barker N, Stange DE, van Es JH, Abo A, Kujala P, Peters PJ, Clevers H (2009). Single Lgr5 stem cells build crypt-villus structures in vitro without a mesenchymal niche. Nature.

[37] Sato T, Clevers H (2013). Primary mouse small intestinal epithelial cell cultures. Methods Mol Biol.

[38] Hoeger B, Diether M, Ballester PJ, Köhn M (2014). Biochemical evaluation of virtual screening methods reveals a cell-active inhibitor of the cancer-promoting phosphatases of regenerating liver. Eur J Med Chem.

[39] Rubio T, Köhn M (2016). Regulatory mechanisms of phosphatase of regenerating liver (PRL)-3. Biochem Soc Trans.

[40] Parry L, Young M, El Marjou F, Clarke AR (2013). Evidence for a crucial role of Paneth cells in mediating the intestinal response to injury. Stem Cells.

[41] Sato T, van Es JH, Snippert HJ, Stange DE, Vries RG, van den Born M, Barker N, Shroyer NF, van de Wetering M, Clevers H (2011). Paneth cells constitute the niche for Lgr5 stem cells in intestinal crypts. Nature.

