# Supplementary figures and images for: The phosphatase PRL-3 affects intestinal homeostasis by altering the crypt cell composition
Source: J Mol Med (Berl). 2021 Jun 15;99(10):1413–26. doi: 10.1007/s00109-021-02097-9 (PMC8455404; doi:10.1007/s00109-021-02097-9)

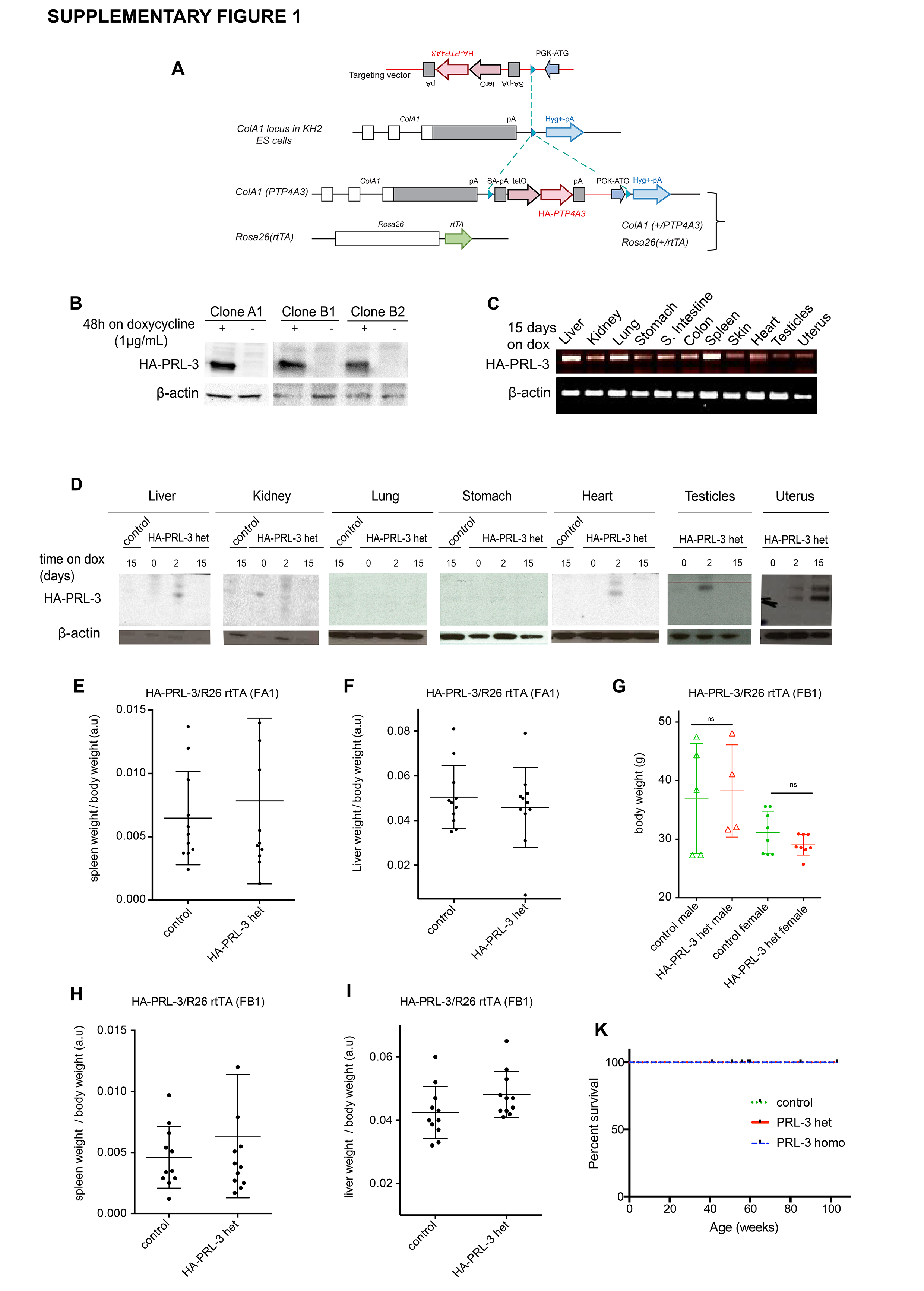

Supplement: Supplementary file 2 — High Resolution Image (TIF 1259 kb) [file 109_2021_2097_MOESM1_ESM.tif]

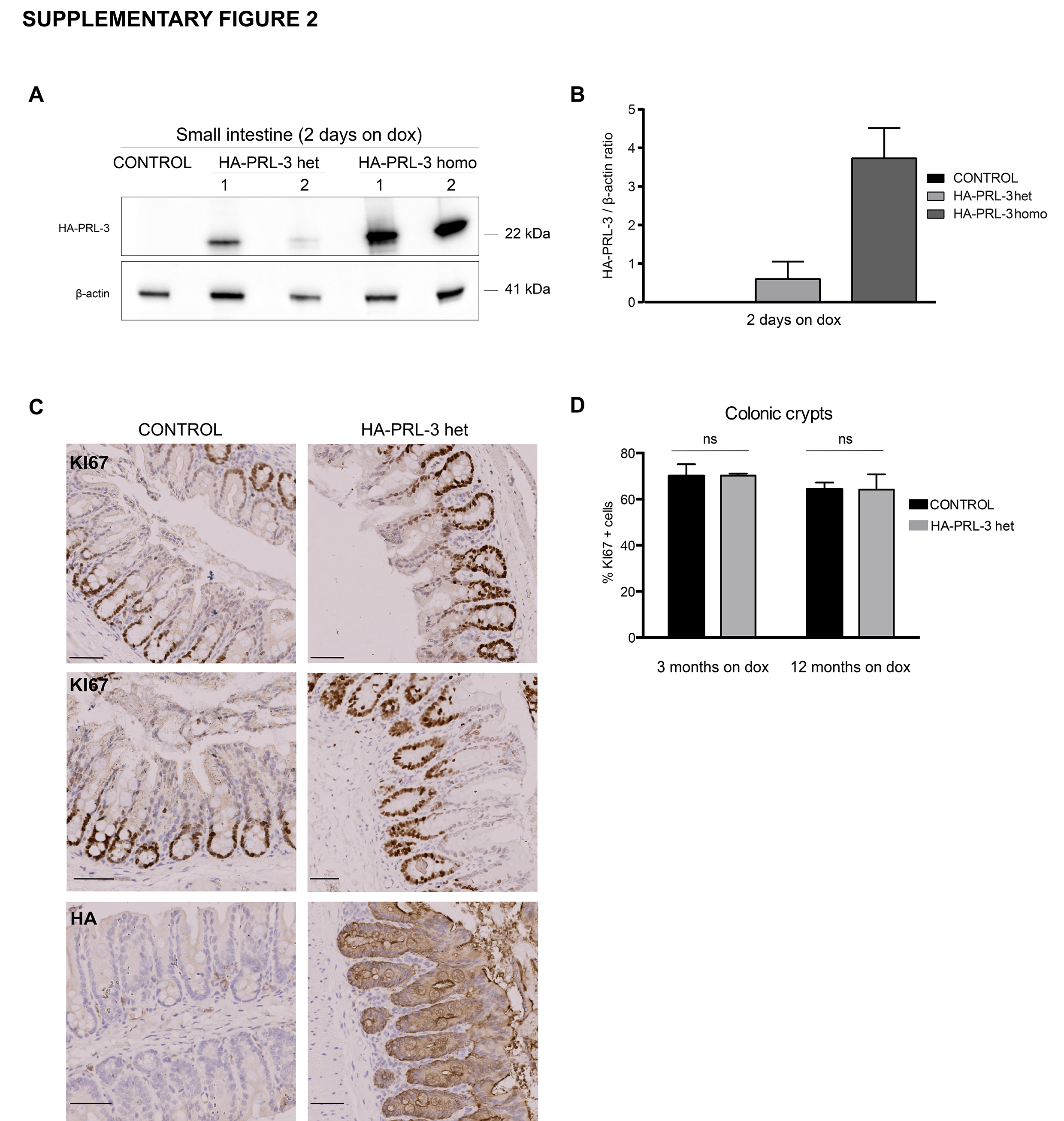

Supplement: Supplementary file 3 — A: Western Blot analysis of small intestine derived tissue samples from HA-PRL-3 wt / R26-rtTA het (control), HA-PRL-3 het/R26-rtTA het (HA-PRL-3 het) and HA-PRL-3 homo / R26-rtTA het (HA-PRL-3 homo) mice treated two days with dox. HA signal was detected using specific antibodies. β actin was used as a loading control. B: Quantification of the Western Blot shown in A. PRL-3 HA expression is presented in relation to β actin loading control (HA-PRL-3 / β actin ratio). HA-PRL-3 het and HA-PRL-3 homo demonstrate the average of sample 1 and 2, respectively. C: Representative images of KI67 staining of colon biopsies from mice treated 3 months with dox upper panel, or 12 months middle panel. The lower panel shows the HA staining for PRL-3 detection in mice after 12 months on dox food. D: Percentage of KI67 positive cells (relative to total cells counted) in the colonic crypts in the different conditions represented in C. 1000 cells were analyzed per tissue sample. Values represent mean ± s.d. of three biological replicates. Statistics: P≤0.1 t-test, compared to control mice. (PNG 2868 kb) [file 109_2021_2097_Fig6_ESM.png]

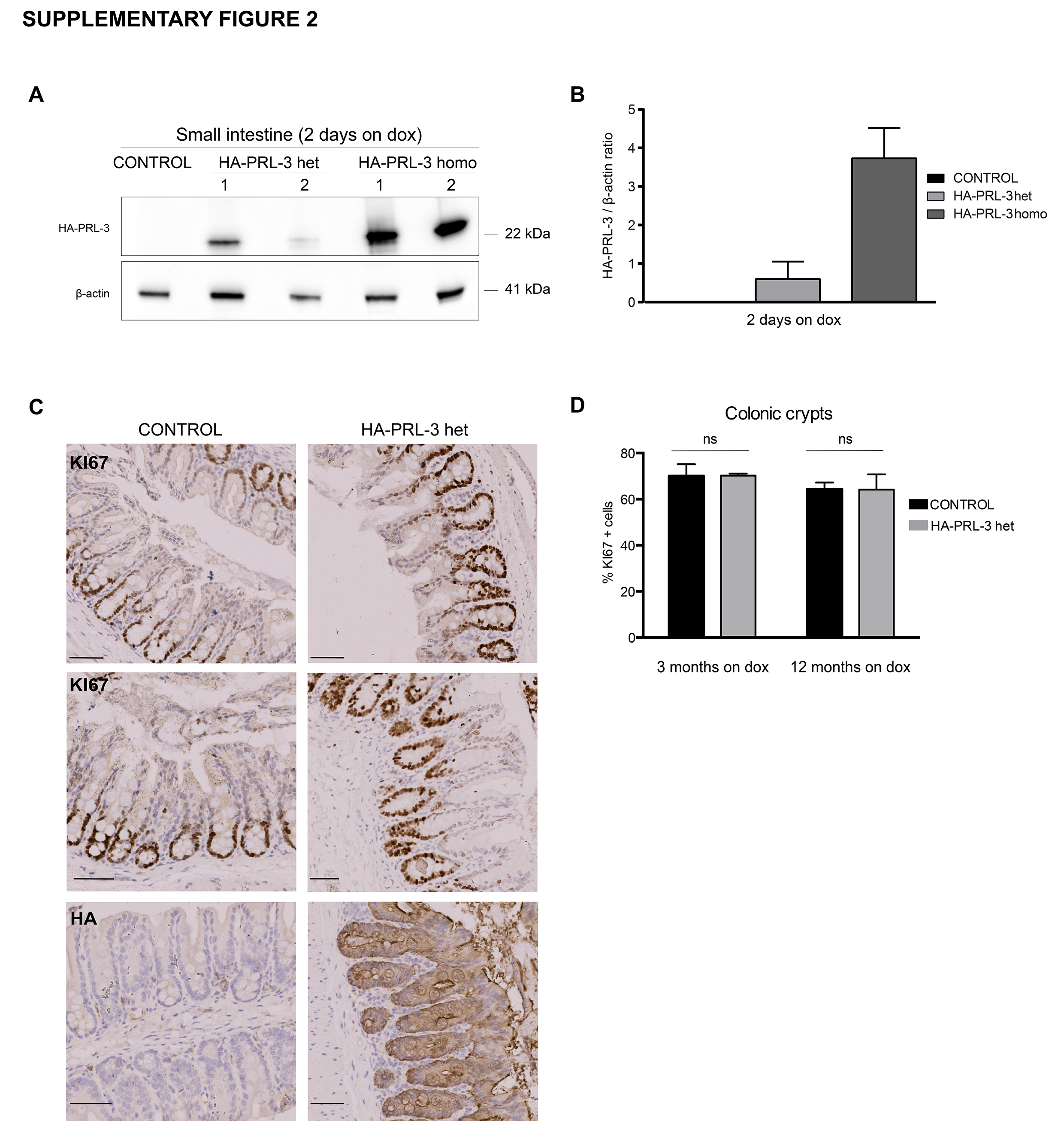

Supplement: Supplementary file 4 — High Resolution Image (TIF 3616 kb) [file 109_2021_2097_MOESM2_ESM.tif]

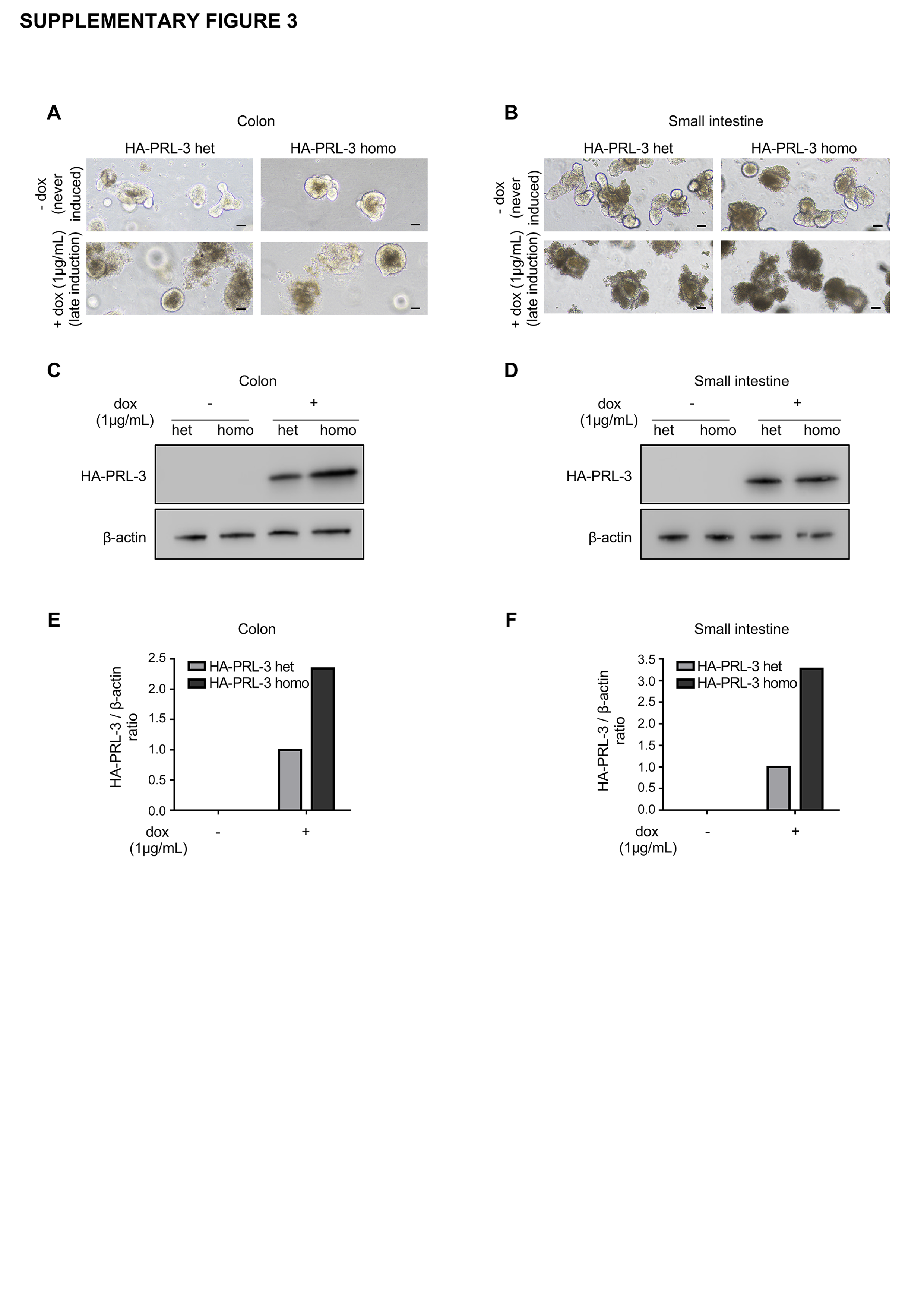

Supplement: Supplementary file 5 — Phenotype and HA-PRL-3 expression of colon (A, C, E) and SI (B, D, F) organoids. 3D cell cultures derived from HA-PRL-3 het / R26-rtTA het mice and HA-PRL-3 homo / R26-rtTA het mice were grown for 2 days after passaging. Subsequently, HA-PRL-3 expression was induced in vitro with 1μg/mL of dox for 2 days (late induction). A, B: Representative bright field inverted microscopy images. Pictures were taken after four days in culture for all conditions. All dox-induced organoids showed the death phenotype compared to non-induced cultures. Scale bar = 50 μm. C, D: Western blot analysis of 3D cell culture after four days. HA signal was detected using specific antibodies. β-actin was used as a loading control. E, F: Quantification of the Western blot shown in C, D. PRL-3 HA expression is presented in relation to β actin loading control (HA-PRL-3 / β actin ratio) and normalized to HA-PRL-3 het induced with dox signal. HA expression in homozygous cultures were increased compared to heterozygous. (PNG 1652 kb) [file 109_2021_2097_Fig7_ESM.png]

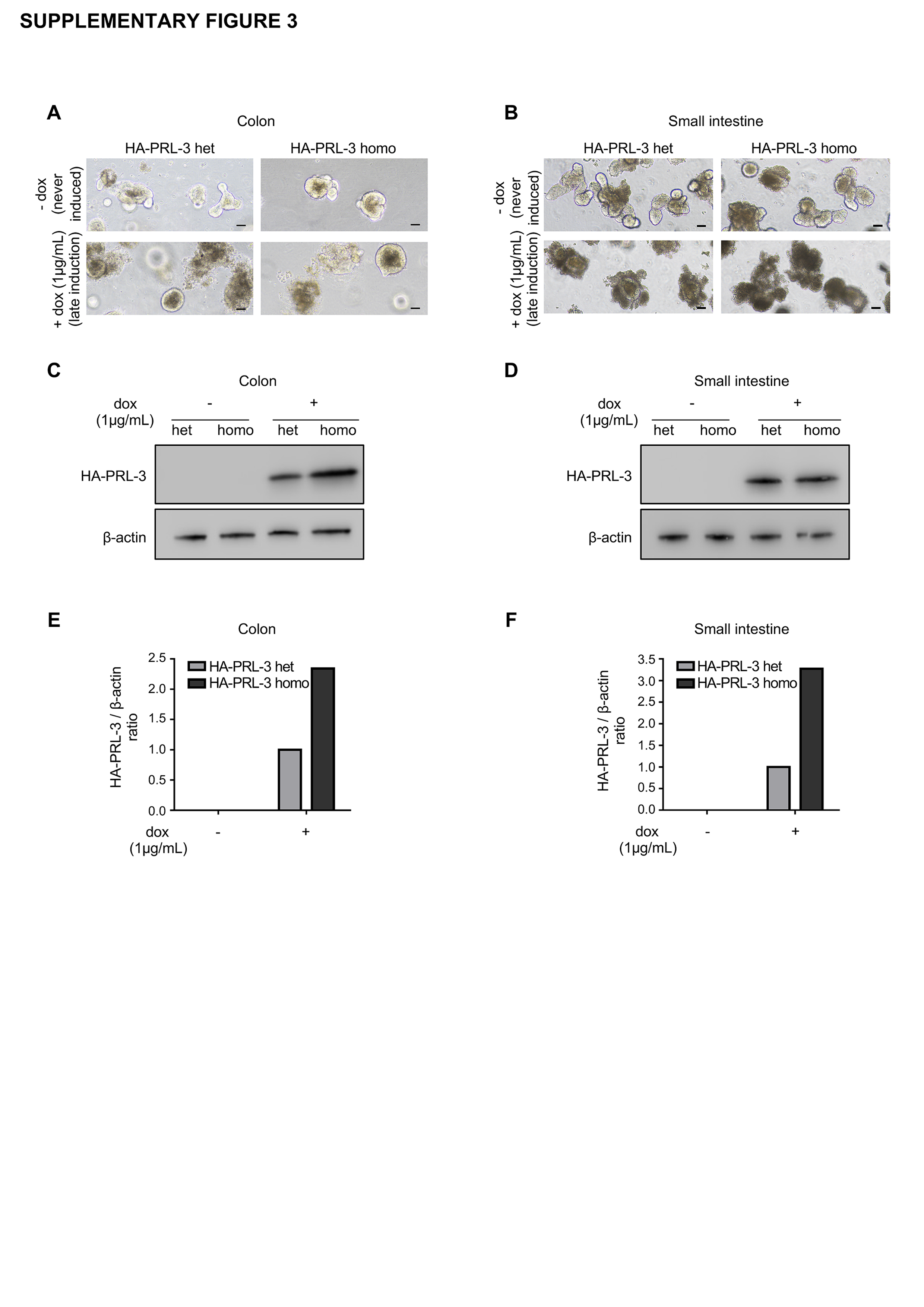

Supplement: Supplementary file 6 — High Resolution Image (TIF 2102 kb) [file 109_2021_2097_MOESM3_ESM.tif]

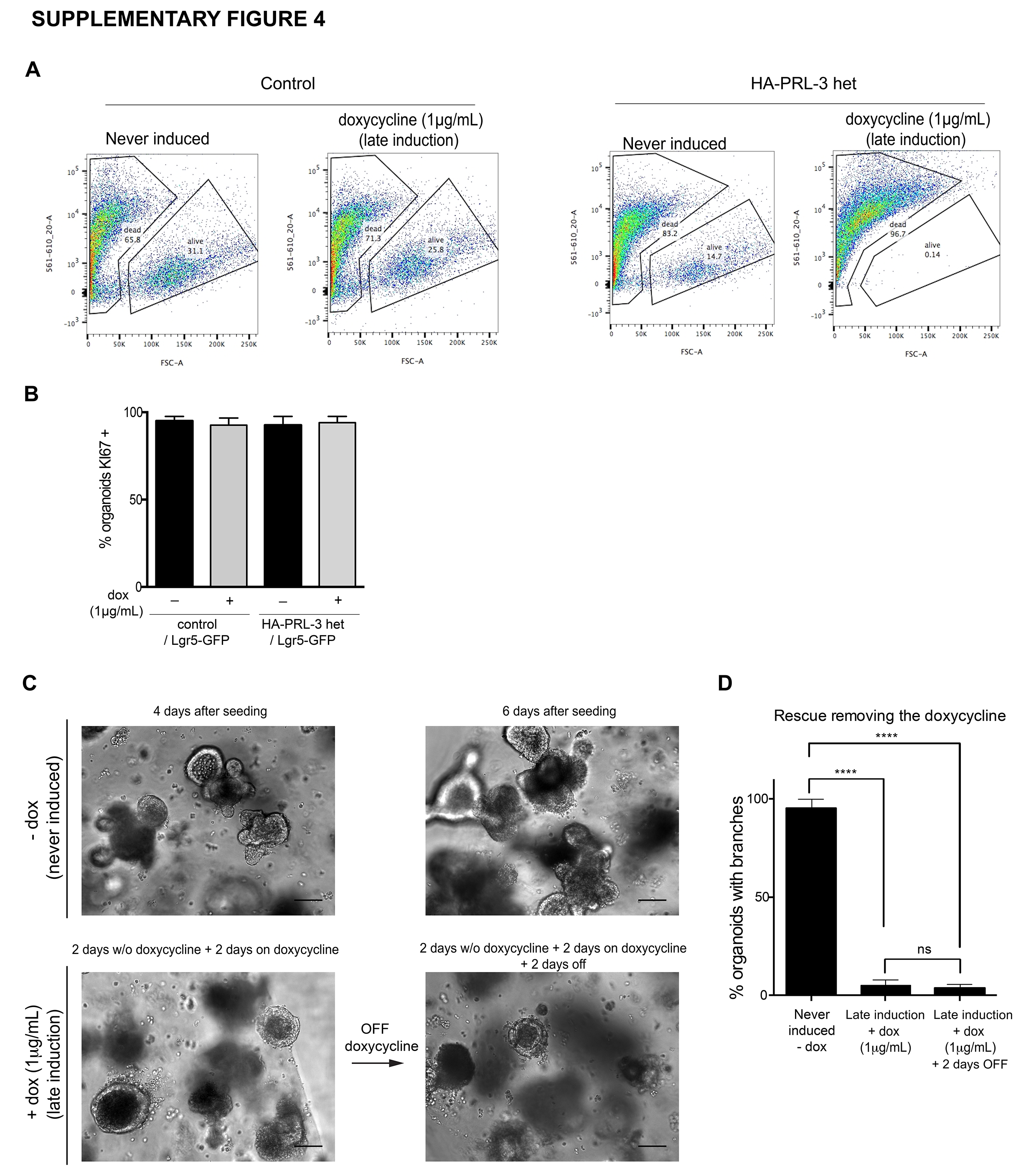

Supplement: Supplementary file 7 — A: PI assay to detect cell death in organoids never induced or late induced. B: Percentage of KI67 positive organoids observed in Fig 4a. The quantification corresponds to the percentage of organoids with any KI67 positive cell with no differences observed between control and PRL-3 het organoids. Although, the pictures in A shows higher number of KI67 positive cells in the control compare to HA-PRL-3 het organoids (with only one or two cells stained for KI67), the number of KI67 positive cells in each organoid was not possible to quantify due to the 3D structure of the organoids. C: Representative bright field inverted microscopy images of small intestinal organoids derived from HA-PRL-3 het mice never induced (no dox) or late induced (dox added in the growth media after 2 days in culture). After 2 days of induction the dox was removed from the media and the organoids were grown in dox free media for two more days. Scale bars: 100 μm. D: Percentage of organoids developed with branches, relative to total organoids, before and after DOX withdrawal. Data are mean ± s.d. N=3, ns: not significant, ****P≤0.0001 ordinary two-tailed one-way ANOVA with Tukey's multiple comparison test. (PNG 2313 kb) [file 109_2021_2097_Fig8_ESM.png]

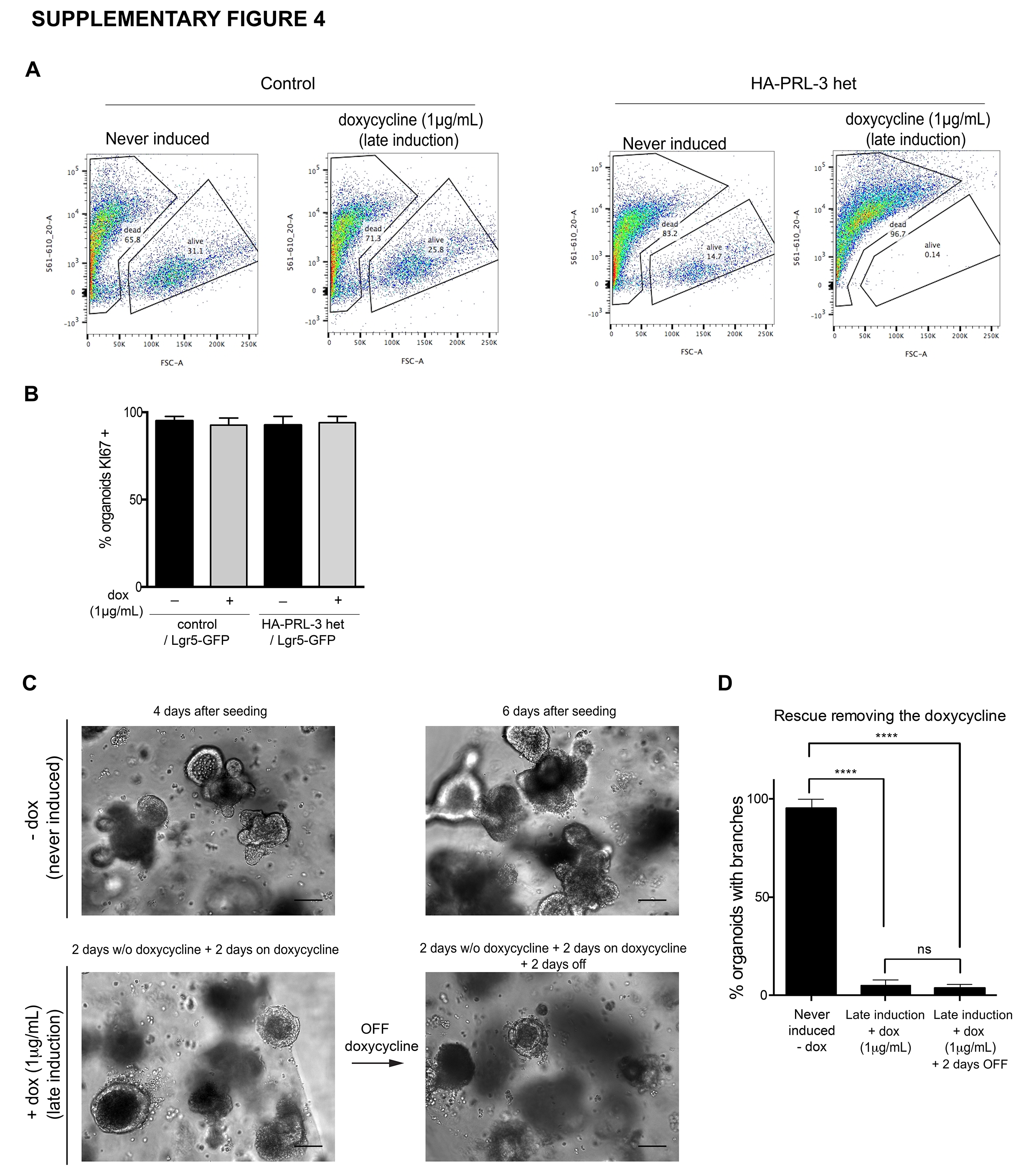

Supplement: Supplementary file 8 — High Resolution Image (TIF 2832 kb) [file 109_2021_2097_MOESM4_ESM.tif]

A

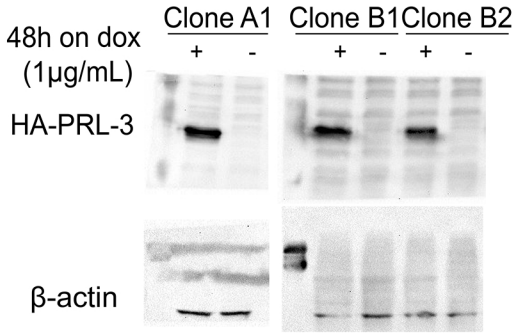

B

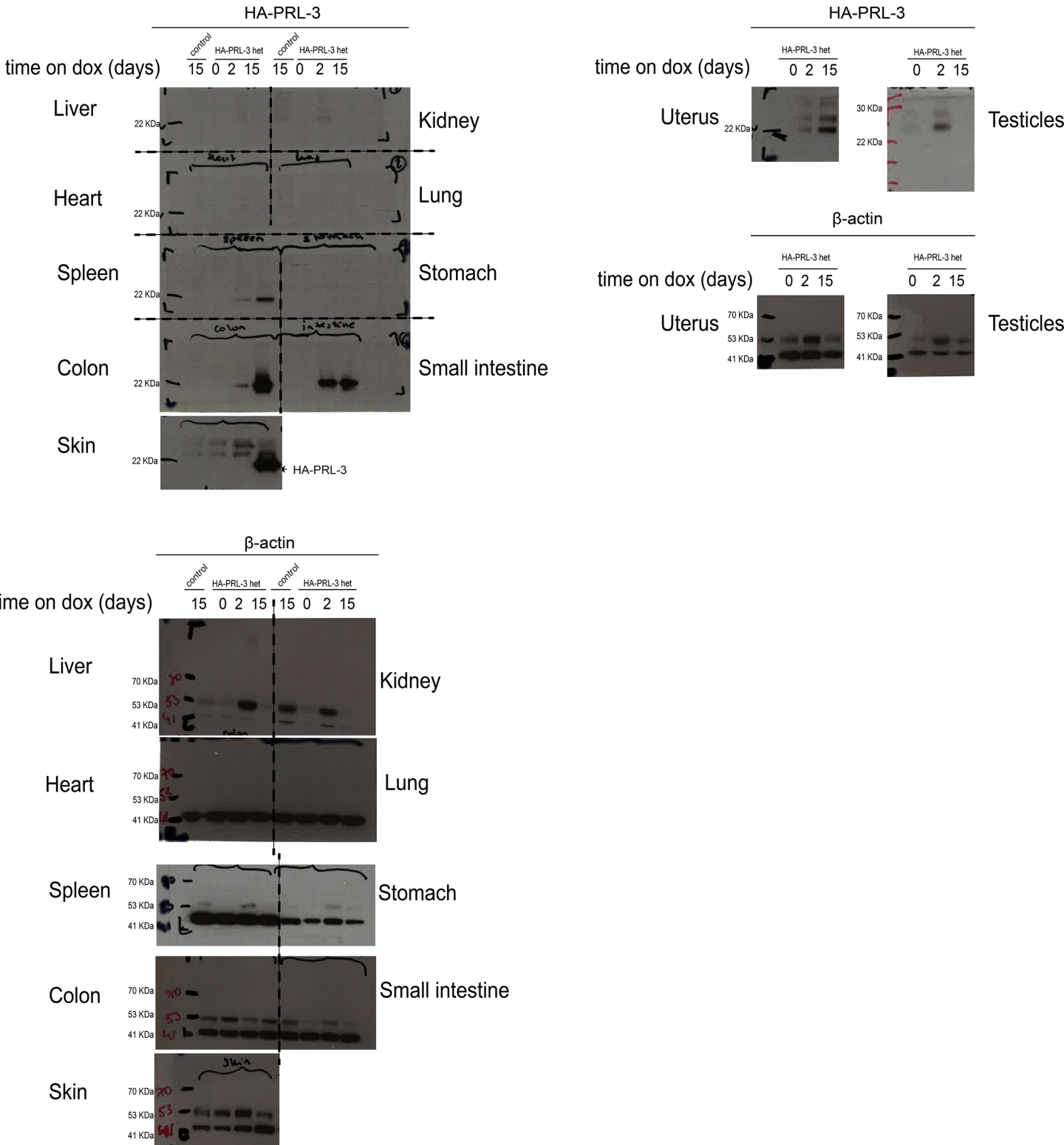

**C**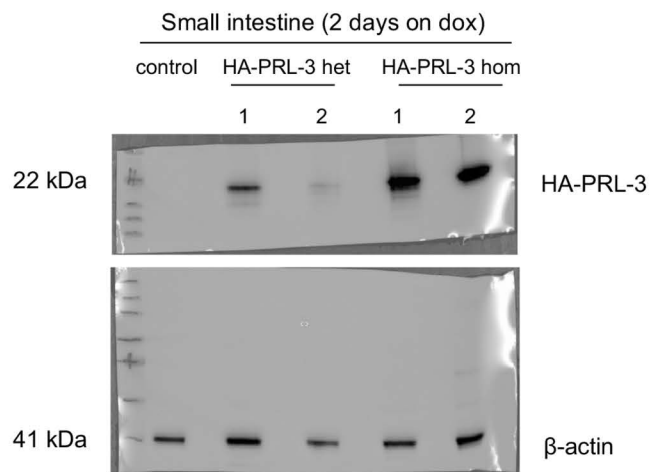**D**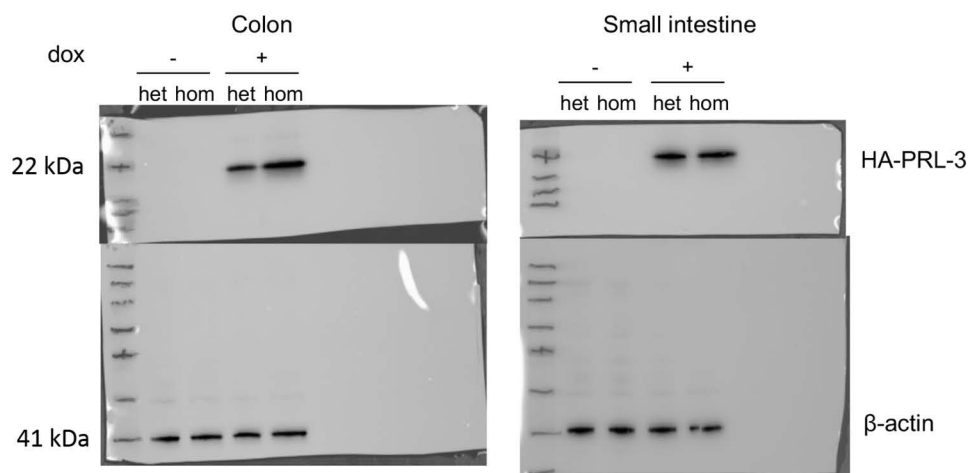

Supplement: Supplementary file 9 — Entire Western blot membranes. A: Entire western blot membranes for the ES clones electroporated with HA-PRL-3 vector using HA antibody and showed in Fig S1B. B: Entire western blot membranes showed in Fig 1a and Fig S1D using tissue samples derived from HA-PRL-3 wt / R26-rtTA het (control) 15 days on dox and HA-PRL-3 het / R26-rtTA het (HA-PRL-3 het) mice fed with dox impregnated pellets for 0 (not treated), 2 or 15 days. Upper membranes correspond to anti HA antibody for HA-PRL-3 detection and lower membranes anti β actin antibody as a loading control. C: Entire western blot membranes showed in Fig S2A using small intestine tissue samples derived from HA-PRL-3 wt / R26-rtTA het (control), HA-PRL-3 het/R26-rtTA het (HA-PRL-3 het) and HA-PRL-3 homo / R26-rtTA het (HA-PRL-3 homo) 2 days on dox. Upper membranes correspond to anti HA antibody for HA-PRL-3 detection and lower membranes correspond to β actin used as a loading control. D: Entire western blot membranes showed in Fig S3C,D. Upper membranes correspond to anti HA antibody for HA-PRL-3 detection and lower membranes anti β actin antibody as a loading control. For further information see Fig S3 caption. (PDF 2433 kb) [file 109_2021_2097_MOESM5_ESM.pdf]
